# Proteinoid Computing on Olivine Substrates

**DOI:** 10.1021/acs.langmuir.6c00952

**Published:** 2026-03-27

**Authors:** Panagiotis Mougkogiannis, Andrew Adamatzky

**Affiliations:** Unconventional Computing Laboratory, 1981 University of the West of England, Coldharbour Lane, Stoke Gifford, Bristol BS16 1QY, U.K.

## Abstract

We investigate proteinoid systems formed on olivine mineral substrates, focusing on self-organization, electrochemical properties, and information-processing capacity. Olivine’s ubiquity in meteorites, planetary surfaces, and protoplanetary disks makes it a geochemically relevant template for prebiotic chemistry across cosmic environments. Glu:Phe:Asp proteinoids synthesized in olivine-rich acidic solutions—mimicking early Earth hydrothermal conditions—were characterized using scanning electron microscopy (SEM), electrochemical impedance spectroscopy (EIS), cyclic voltammetry (CV), and differential pulse voltammetry (DPV). The proteinoids self-assembled into spherical microspheres (2–15 μm in diameter), dendritic networks, and complex mineral-templated architectures. Budding-like reproduction and neuron-like branching morphologies emerged spontaneously. Electrochemical analysis revealed stable impedance profiles that, when thresholded, enabled Boolean logic operations (AND, OR, XOR, and NOT). Galvanostatic measurements showed spontaneous electrical oscillations with burst dynamics, heavy-tailed distributions, and non-Poissonian statistics, which are signatures of complex adaptive systems. Olivine substrates stabilized the electrical behavior while preserving computational functionality. These findings suggest that proteinoid–olivine hybrids can perform unconventional computing tasks while simultaneously exhibiting biomimetic self-assembly and primitive reproductive behaviors. This work illuminates mineral–organic interactions relevant to both terrestrial and extraterrestrial prebiotic chemistry and provides a foundation for bioinspired computing systems that merge organic self-organization with mineral-based information processing.

## Graphical abstract

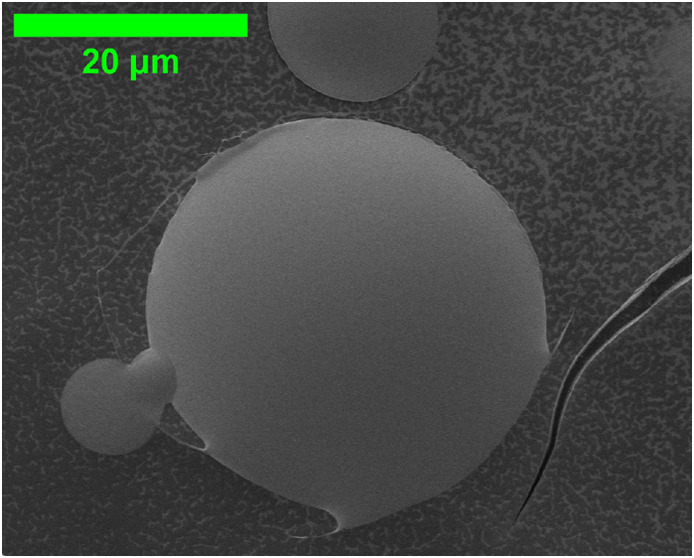

## Introduction

Sidney Fox’s pioneering work on thermal proteinoids demonstrated that heating amino acid mixtures to 130–200 °C under dry conditions leads to spontaneous polymerization, forming proteinoids that self-assemble into hollow, membrane-bounded microspheres.^1–3^ These protocells generate action-potential-like responses^4^ and reproduce by budding,^5,6^ suggesting rudimentary information processing and replication.^7–11^ Recent studies have renewed interest in proteinoid systems, demonstrating catalytic activity, metabolic features, and information storage potential,^8,9,12–25^ while geological evidence indicates that early Earth hydrothermal environments render thermal synthesis geochemically plausible.^26,27^ Olivine (Mg,Fe)_2_SiO_4_ is among the most abundant minerals in the universe (Figure 1),^28^ occurring in meteorites,^29^ planetary surfaces,^30^ asteroids,^31,32^ and protoplanetary disks. In aqueous environments, olivine dissolves via surface-controlled mechanisms, releasing divalent cations (Mg^2+^, Fe^2+^) that form stable complexes with amino acids and peptides.^33–36^ Serpentinization—the hydrothermal alteration of olivine—generates alkaline and reducing conditions favorable for abiotic organic synthesis,^37–39^ while olivine surfaces promote amino acid polymerization through adsorption and templating effects.^40,41^ Proteinoid systems exhibit structural diversity, forming architectures from uniform microspheres (1–50 μm) to dendritic networks.^27,42–44^ Mineral substrates influence proteinoid organization through surface chemistry, charge distribution, and nucleation sites.^45,46^ Self-assembly follows classical nucleation theory driven by hydrogen bonding, π–π stacking, metal coordination, and structured water,^47–50^ responding sensitively to pH, ionic strength, temperature, and mineral surfaces.^51,52^ Electrochemical studies reveal that proteinoids possess complex electrical properties with impedances ranging from kilo-ohms to mega-ohms, exhibiting nonideal capacitive behavior.^53–56^ Carboxylate groups coordinate metal ions and participate in proton-coupled electron transfer, while aromatic residues contribute to π-electron delocalization and conductive pathways.^57–60^ Hydration dramatically enhances conductivity,^61,62^ and recent findings suggest memristive behavior characterized by history-dependent resistance.^63–65^ Chemical logic operations in soft matter implement Boolean logic through pH modulation, redox switching, and impedance thresholding.^66–68^ DNA-based systems,^69,70^ oscillatory reactions,^71,72^ excitable media,^73,74^ and memristive materials^75,76^ demonstrate computational potential in chemical systems, establishing a conceptual framework for proteinoid–olivine logic operations. Oscillatory electrochemical behavior bridges nonequilibrium thermodynamics and biological information processing,^77–79^ with burst dynamics and heavy-tailed distributions characteristic of biochemical networks.^80–85^ The oscillatory fluctuations observed in proteinoid–olivine systems suggest self-organizing processes linking prebiotic chemistry to emergent biological complexity.^86^ Mineral-templated assembly plays a critical role in prebiotic chemistry. Clay minerals adsorb amino acids, facilitate peptide bond formation, and protect polymers,^87–90^ while metal cations promote polymerization through coordination complexes.^91–94^ Modern biomineralization offers insight into mineral–organic interactions through templating functions.^95–102^ Dendritic patterns arise in abiotic systems through metal electrodeposition and thermal gradients,^103,104^ following diffusion-limited aggregation with fractal dimensions between 1.7 and 2.5,^105–110^ explained by self-organized criticality.^111–119^ Protocellular reproduction mechanisms include lipid vesicle division,^40^ coacervate budding,^120,121^ and proteinoid budding,^5,122^ explained by Rayleigh–Plateau instability.^123–127^ Successful division requires coordinated membrane growth, content replication, and physical fission,^128–131^ enabled by autocatalytic networks.^132,133^ The transition from abiotic chemistry to cellular life involves compartmentalization, catalytic networks, and information storage.^134–138^ Physical computing in biological materials exploits intrinsic information-processing capabilities. Slime molds solve shortest-path problems,^139,140^ fungal mycelia transmit signals for distributed decision-making,^141–144^ and reservoir computing leverages complex temporal dynamics.^145–148^ Biological materials encode memory through structural and chemical states,^149–152^ inspiring neuromorphic systems and memristive devices.^153–156^ Prebiotic chemistry in mineral-rich environments examines mineral–organic interactions facilitating increasing complexity. Hydrothermal synthesis generates biomolecules from simple precursors,^157,158^ iron–sulfur minerals catalyze carbon fixation pathways,^159,160^ and transition-metal ions catalyze peptide formation,^91,161–163^ with amino-acid thermal stability varying significantly.^164–168^ Laboratory simulations demonstrate abiotic synthesis under geochemically realistic early Earth conditions.^169–172^ This work explores how olivine-templated proteinoid systems exhibit complex behaviors ranging from neuron-like morphologies to Boolean logic operations that may illuminate the chemical foundations preceding biological information processing.

**Figure 1:**
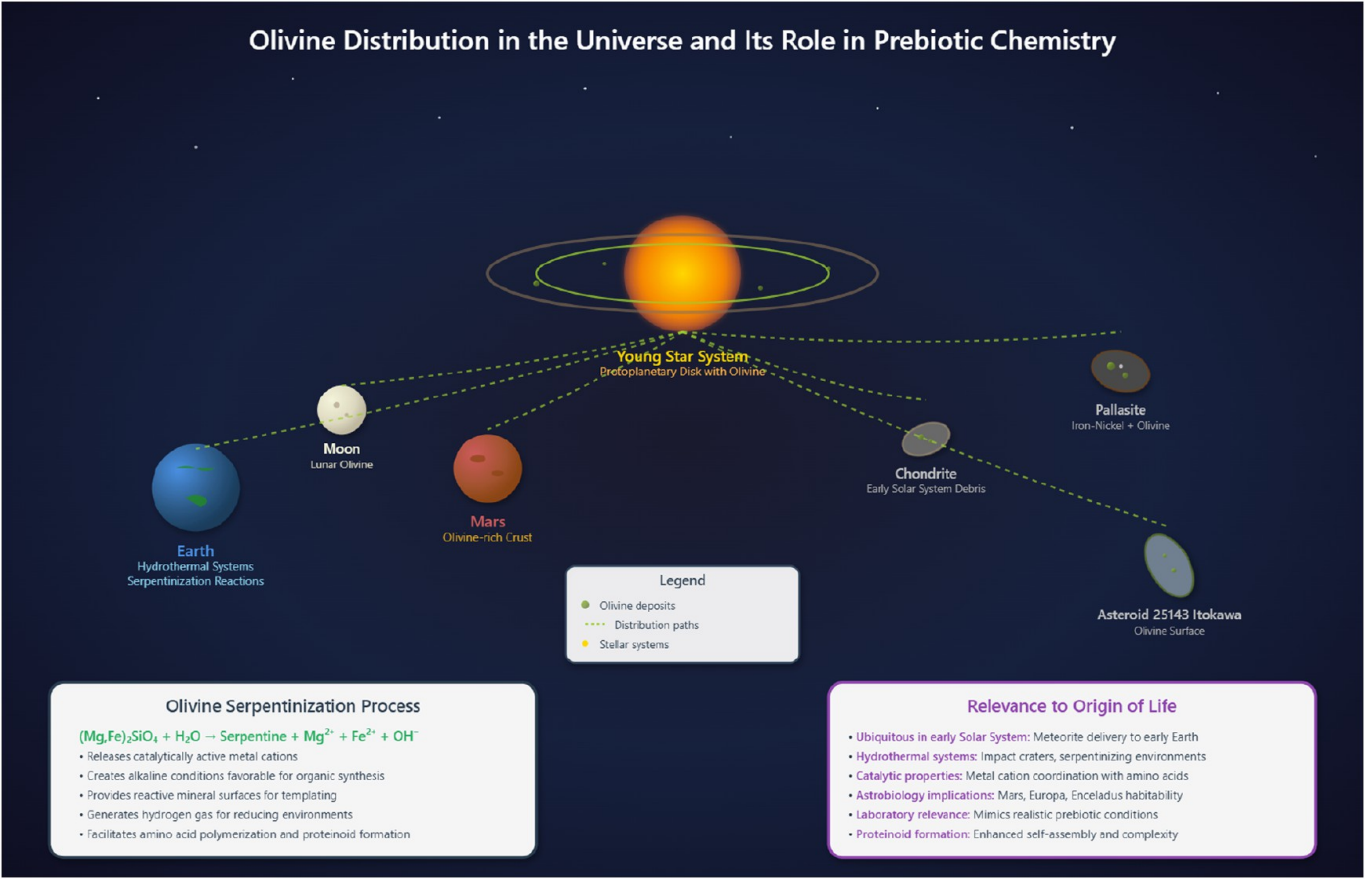
Olivine (Mg,Fe)_2_SiO_4_ is ubiquitous in cosmic environments, including protoplanetary disks, meteorites, planetary surfaces, and asteroids. Its widespread presence makes it a geochemically significant mineral for origin-of-life studies. In aqueous conditions, olivine undergoes serpentinization—a reaction with water that releases divalent metal cations (e.g., Mg^2+^, Fe^2+^) and generates alkaline environments. This process not only alters geochemical conditions but also provides reactive surfaces that can facilitate the formation of prebiotic organic compounds. Due to its catalytic properties and cosmic abundance, olivine is an ideal mineral for investigating prebiotic chemistry under early planetary conditions. Its role in mineral–organic interactions offers insight into plausible pathways for the emergence of life’s molecular precursors on both Earth and other potentially habitable worlds.

## Methods and Materials

### Proteinoid Synthesis

Proteinoids were synthesized using a modified thermal polymerization method based on the protocol developed by Fox and Harada, incorporating olivine into the system. Equimolar quantities of l-glutamic acid, l-phenylalanine, and l-aspartic acid (Sigma-Aldrich, >99% purity) were combined in a 1:1:1 molar ratio to prepare the Glu:Phe:Asp proteinoid system. A total of 10 g of the amino acid mixture was placed in a round-bottom flask and heated to 180 °C under a nitrogen atmosphere for 6 h to prevent oxidative degradation. Thermal polymerization was carried out using a heating mantle equipped with temperature control. Continuous stirring was applied to ensure uniform heat distribution and to prevent localized overheating. Following the polymerization period, the resulting material was cooled to room temperature under nitrogen. The process yielded a brown, glass-like proteinoid polymer, which was subsequently ground into a fine powder using a mortar and pestle. The proteinoid powder was stored under desiccated conditions at 4 °C until further use. To form microspheres, 100 mg of proteinoid powder was dissolved in 10 mL of boiling distilled water. The solution was then allowed to cool gradually to room temperature over a period of 2 h. This controlled cooling promoted the self-assembly of spherical structures with diameters ranging from 2 to 15 μm, as confirmed by optical microscopy.

Scanning electron microscopy (SEM) imaging was performed using a FEI ESEM Quanta 450 FEG instrument. This versatile microscope operates in three modes: high vacuum (HV), low vacuum (LV), and environmental SEM (ESEM). High vacuum mode was used when conventional specimen preparation and conductive coating were required. Low vacuum mode enabled imaging of nonconductive samples without the need for additional conductive layers such as carbon or gold. The thermally assisted field emission gun (FEG) provided high beam brightness and resolution, allowing detailed visualization of the sample morphology.

### Olivine Crystal Integration

Natural olivine crystals (forsterite–fayalite solid solution, (Mg,Fe)_2_SiO_4_) were crushed and sieved to obtain particles in the size range of 100–500 μm. The particles were subsequently cleaned by sonication in distilled water for 30 min and then dried at 60 °C overnight. To prepare the proteinoid–olivine hybrid system, 1 g of olivine particles was added to the proteinoid solution during the cooling phase of microsphere formation. This timing facilitated mineral-templated assembly. An acid-treated olivine solution was prepared by dispersing olivine particles in 0.1 M HCl (pH 2.0) and stirring the suspension for 24 h to achieve partial mineral dissolution and surface activation. This treatment resulted in the release of Mg^2+^ and Fe^2+^ cations into the solution, while generating reactive silanol groups on the olivine surface. These functional groups support coordination interactions with carboxylate and amino groups present in the proteinoids. The resulting olivine–proteinoid suspensions were allowed to age for 48 h at room temperature under gentle agitation to facilitate equilibration and the completion of hybrid microsphere assembly.

### Data Acquisition with PicoLog ADC-24

Electrochemical data were collected using a PicoLog ADC-24 data logger (Pico Technology), featuring 24-bit analog-to-digital conversion and a sampling rate of 1 Hz. The ADC-24 was selected for its high resolution (effective number of bits >20) and low noise characteristics, which are essential for detecting subtle electrochemical signals in high-impedance biological systems (Figure 2). Temperature compensation was achieved using the built-in cold junction functionality for thermocouple readings. Differential input channels were employed to minimize common-mode noise and electromagnetic interference. Input ranges were configured based on the expected signal amplitudes: ±2.5 V for impedance measurements and ±39 mV for monitoring low-amplitude spontaneous potential fluctuations. Sampling synchronization was maintained using the device’s internal crystal oscillator, which provides timing accuracy better than 0.01%, ensuring precise temporal alignment for time-series analysis. Data streams were recorded continuously using PicoLog 6 software, which automatically segmented files every 24 h to facilitate efficient data management and postprocessing. Extended measurement sessions, lasting up to 93,000 s, required careful control of environmental variables. The experimental setup was housed in a temperature-controlled enclosure equipped with vibration damping to ensure thermal stability and minimize mechanical noise.

**Figure 2:**
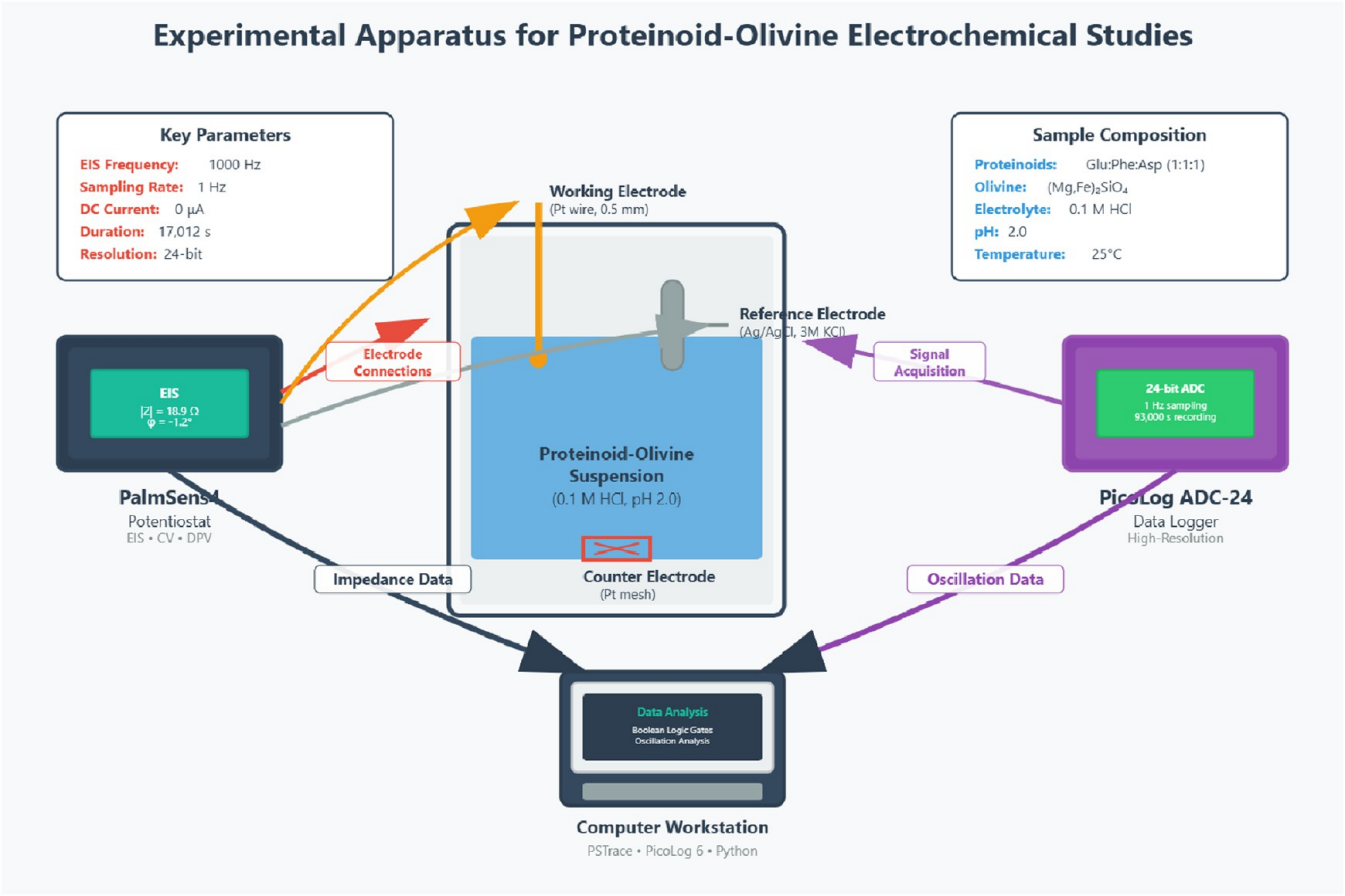
Experimental setup employs a three-electrode electrochemical cell containing a proteinoid–olivine suspension. The medium consists of Glu:Phe:Asp proteinoids dissolved in 0.1 M HCl at pH 2.0. The electrodes include a platinum wire working electrode (diameter 0.5 mm), an Ag/AgCl reference electrode (3 M KCl), and a platinum mesh counter electrode. Electrochemical measurements are performed using a PalmSens4 potentiostat/galvanostat. The system supports impedance spectroscopy (EIS), cyclic voltammetry (CV), and differential pulse voltammetry (DPV). For monitoring long-term spontaneous potential oscillations, a PicoLog ADC-24 data logger is employed, offering 24-bit resolution and a sampling rate of 1 Hz. The PalmSens system operates at a fixed frequency of 1000 Hz for impedance measurements, using a galvanostatic mode with zero DC bias current. This configuration enables continuous acquisition of electrochemical data over extended durations, up to 17,012 s. Data from both instruments are synchronized and processed on a computer using PSTrace and PicoLog 6 software, alongside custom Python scripts. These scripts implement Boolean logic operations, characterize oscillatory behavior, and perform statistical analysis. Color-coded wiring distinguishes electrode and signal paths: red for the counter electrode, orange for the working electrode, gray for the reference electrode, purple for PicoLog input, and dark tones for digital data flow. This integrated system allows simultaneous measurement of impedance-based logic operations, spontaneous electrochemical oscillations, and dynamic behavior of proteinoid–mineral hybrid structures, offering comprehensive insight into their computational capabilities.

### Impedance Spectroscopy with PalmSens

Electrochemical impedance spectroscopy (EIS) measurements were performed using a PalmSens4 potentiostat galvanostat (PalmSens, Alvatec UK), equipped with an EIS module for frequency response analysis. The system was operated in galvanostatic mode with zero DC current (*I*_DC_ = 0 μA) and an AC perturbation amplitude of 10 μA_RMS_, over a frequency range of 0.1 Hz to 100 kHz. A three-electrode configuration was employed, consisting of a platinum wire working electrode (0.5 mm diameter), a platinum mesh counter electrode, and an Ag/AgCl reference electrode (3 M KCl). The working electrode was positioned 2 mm above the proteinoid–olivine sample to maintain a consistent distance and enable unhindered diffusion. Impedance measurements were conducted at fixed frequency intervals, with a primary focus at 1000 Hz for time-scan experiments. The total measurement duration was 17,012 s, enabling the observation of long-term electrochemical variations. Prior to each experimental session, the PalmSens system was calibrated using standard dummy cells with known resistance and capacitance values. Data acquisition was carried out using PSTrace software, which recorded impedance magnitude (|*Z*|), phase angle (ϕ), and real (*Z*′) and imaginary (*Z*″) components at 1-s intervals. To compensate for temperature-induced drift, regular baseline measurements were recorded in electrolyte solutions lacking sample material.

### Voltammetric Characterization Protocols

Cyclic voltammetry (CV) and differential pulse voltammetry (DPV) experiments were conducted using the PalmSens4 system, configured with optimized parameters for biological sample analysis. CV was performed over a potential range of −0.5 to +0.5 V vs Ag/AgCl, at a scan rate of 100 mV/s, with 100 consecutive cycles recorded. This protocol allowed evaluation of electrochemical conditioning and system stability. The selected potential window was chosen to avoid water electrolysis while capturing redox activity associated with amino acid residues and metal coordination processes. DPV measurements were carried out using pulse amplitudes ranging from 0.1 to 1.0 V, with pulse widths of 50 ms and step potentials of 5 mV to optimize resolution and sensitivity. Between measurements, the working electrode was cleaned by potential cycling in 0.5 M H_2_SO_4_ followed by rinsing with distilled water. All voltammetric experiments were conducted in stirred solutions, after purging with nitrogen for 10 min to remove dissolved oxygen. The electrolyte consisted of an olivine acid solution (0.1 M HCl, pH 2.0) containing dissolved proteinoid material at a concentration of 1 mg/mL. Additional experiments were performed using 0.1 M KCl as a supporting electrolyte to distinguish between specific proteinoid–olivine interactions and general ionic conductivity effects. Python scripts incorporating the scipy and numpy libraries were used for statistical analysis, peak detection, and frequency domain transformations. Boolean logic gates were implemented using impedance magnitude thresholding at 0.14 kΩ. Consecutive binary values served as logic inputs for the realization of AND, OR, XOR, NAND, NOR, and NOT operations. Oscillatory behavior was analyzed statistically through the computation of amplitude distributions, interpeak intervals, and frequency spectra. Fast Fourier Transform (FFT) algorithms were applied to obtain spectral characteristics. To assess non-Poissonian behavior, Kolmogorov–Smirnov (K–S) testing and chi-squared (χ^2^) goodness-of-fit analysis were employed.

## Results and Discussion

### Hierarchical Self-Assembly and Morphological Diversity in Olivine-Mediated Proteinoid Systems

Scanning electron microscopy reveals remarkable morphological diversity in proteinoid systems incubated in olivine acidic solutions. Glu:Phe:Asp proteinoids form structures ranging from spherical microspheres to dendritic networks resembling early neural architectures. Under acidic conditions (pH = 2.0), olivine dissolution creates a dynamic chemical environment by releasing catalytically active Mg^2+^ and Fe^2+^ cations and generating reactive silanol surface groups. These conditions shape the thermodynamics and kinetics of proteinoid self-assembly, yielding mineral-templated structures with diameters ranging from 2–25 μm and exhibiting striking structural complexity. Some assemblies display budding-like behavior reminiscent of cellular division, while others form interconnected tubular networks suggestive of primitive compartmentalization. Branching morphologies frequently echo the fractal geometries observed in biological neural dendrites. This morphological plasticity reflects a strong sensitivity to localized chemical gradients, mechanical stress, and surface-mediated interactions. The coupling of organic self-assembly with inorganic catalytic surfaces may recapitulate foundational processes underlying biological organization. SEM analysis provides quantitative insight into size distributions, network connectivity, and assembly pathways, thereby bridging simple abiotic chemistry and complex living morphologies. Olivine interacts with Glu:Phe:Asp proteinoids through hydrolysis, metal coordination, and surface-templating reactions that alter both mineral surface chemistry and proteinoid assembly behavior. Olivine dissolution in acidic aqueous environments proceeds via hydrolysis (eqs 1 and 2).

1 $$\mathrm{Forsterite}\;\mathrm{component}:\;{\mathrm{Mg}}_{2}{\mathrm{SiO}}_{4}+4H^{+}\to 2{\mathrm{Mg}}^{2+}+H_{4}{\mathrm{SiO}}_{4}$$

2 $$\mathrm{Fayalite}\;\mathrm{component}:\;{\mathrm{Fe}}_{2}{\mathrm{SiO}}_{4}+4H^{+}\to 2{\mathrm{Fe}}^{2+}+H_{4}{\mathrm{SiO}}_{4}$$

In acidic conditions like those during olivine dissolution (pH 2–4), silicic acid mainly exists as monomers. The divalent cations released are available to coordinate with proteinoid functional groups. The dissolution kinetics follow a surface-controlled mechanism where the rate depends on the proton activity (eq 3).

3 $${\mathrm{Rate}}_{\mathrm{dissolution}}=k\cdot a_{H^{+}}^{n}\cdot \mathrm{SA}$$

where *k* is the rate constant, *a*_H^+^_ is proton activity, *n* is the reaction order (typically 0.5–1.0 for olivine), and SA is the specific surface area. The released Mg^2+^ and Fe^2+^ cations strongly interact with the carboxylate groups found in glutamic and aspartic acid residues in the proteinoids. This happens through coordination chemistry (eqs 4–6).

4 $$\mathrm{Magnesium}\;\mathrm{coordination}:\;{\mathrm{Mg}}^{2+}+2\mathrm{Glu}‐{\mathrm{COO}}^{-}\to [\mathrm{Mg}\left(\mathrm{Glu}‐\mathrm{COO}{)}_{2}\right]$$

5 $$\mathrm{Iron}\;\mathrm{coordination}:\;{\mathrm{Fe}}^{2+}+2\mathrm{Asp}‐{\mathrm{COO}}^{-}\to [\mathrm{Fe}\left(\mathrm{Asp}‐\mathrm{COO}{)}_{2}\right]$$

6 $$\mathrm{Mixed}\;\mathrm{coordination}:\;{\mathrm{Mg}}^{2+}+\mathrm{Glu}‐{\mathrm{COO}}^{-}+\mathrm{Asp}‐{\mathrm{COO}}^{-}\to \left[\mathrm{Mg}\left(\mathrm{Glu}‐\mathrm{COO}\right)\left(\mathrm{Asp}‐\mathrm{COO}\right)\right]$$

These coordination complexes act as cross-linking points. They can greatly change how proteinoids assemble themselves. The formation constants for these complexes are important. The log *K* values usually range from 3 to 5 for carboxylate–metal coordination (eq 7).

7 $$K_{\mathrm{formation}}=\frac{[M\left(\mathrm{COO}{)}_{2}\right]}{[M^{2+}]{\left[{\mathrm{COO}}^{-}\right]}^{2}}$$

Silicic acid from olivine dissolution can condense. This forms silicate networks that connect with the proteinoid matrix (eqs 8 and 9).

8 $$\mathrm{Silanol}\;\mathrm{formation}:\;H_{4}{\mathrm{SiO}}_{4}⇌H_{3}{\mathrm{SiO}}_{4}^{-}+H^{+}$$

9 $$\mathrm{Siloxane}\;\mathrm{bridging}:\;2H_{3}{\mathrm{SiO}}_{4}^{-}\to H_{2}{\mathrm{Si}}_{2}O_{7}^{2-}+H_{2}O$$

Amino groups in proteinoids can bond with silicate species through hydrogen bonds and electrostatic interactions (eq 10).

10 R‐NH3++H3SiO4−→R‐NH3+···OSiH3O3−

The phenylalanine residues are not directly involved in metal coordination. Still, they are key in the assembly process. They help through π–π stacking interactions, which are influenced by metal-carboxylate cross-links (eq 11).

11 $$\mathrm{Phe}‐\mathrm{Phe}\;\mathrm{stacking}:\pi_{\mathrm{Phe}}+\pi_{\mathrm{Phe}}\to \pi \text{−}\pi \;\mathrm{complex}$$

The combination of metal coordination and π–π stacking forms a hybrid organic–inorganic network. In this network, self-assembly thermodynamics are controlled by the sum of individual interaction energies (eq 12).

12 $$\Delta G_{\mathrm{assembly}}=\Delta G_{\mathrm{hydrophobic}}+\Delta G_{\mathrm{metal}‐\mathrm{coord}}+\Delta G_{\mathrm{electrostatic}}+\Delta G_{\mathrm{silicate}‐\mathrm{binding}}$$

The oxidation of Fe^2+^ to Fe^3+^ happens when oxygen is dissolved. This process can create more complexity. It forms iron oxides and hydroxides, which act as nucleation sites (eqs 13 and 14).

13 $$\mathrm{Iron}\;\mathrm{oxidation}:\;4{\mathrm{Fe}}^{2+}+O_{2}+4H^{+}\to 4{\mathrm{Fe}}^{3+}+2H_{2}O$$

14 $$\mathrm{Hydroxide}\;\mathrm{precipitation}:\;{\mathrm{Fe}}^{3+}+3H_{2}O\to \mathrm{Fe}(\mathrm{OH}{)}_{3}+3H^{+}$$

The proteinoid carboxylate groups can change the pH buffering capacity. This affects precipitation reactions and creates pH gradients, which influence where mineral phases are located (eq 15).

15 $$\mathrm{pH}\;\mathrm{buffering}:\;\mathrm{RCOOH}+{\mathrm{OH}}^{-}⇌{\mathrm{RCOO}}^{-}+H_{2}O$$

The interplay of dissolution, coordination, and precipitation reactions creates a dynamic chemical environment in which proteinoid self-assembly occurs alongside evolving mineral surfaces, metal-cation gradients, and silicate networks. This coupling yields hybrid structures with enhanced stability and novel morphologies, potentially recapitulating organic–inorganic coevolution in prebiotic olivine-rich environments. Scanning electron microscopy of Glu:Phe:Asp proteinoids incubated in olivine acidic solutions reveals diverse self-assembled morphologies that vary widely in size and structural complexity (Figure 3). Spherical microspheres (panels a–d, 2–15 μm in diameter) represent the fundamental mode of proteinoid assembly originally described by Fox. Their spherical symmetry and smooth surfaces indicate thermodynamic stabilization through surface-energy minimization, driven by hydrophobic collapse of phenylalanine residues and electrostatic interactions among glutamic and aspartic acid groups. Paired microspheres (panel a) demonstrate budding reproduction, a key behavior linking abiotic polymer chemistry to primitive cellular division. The olivine acidic environment provides optimal pH and ionic strength to support structural integrity during this process. Networked structures (panels e–h) exhibit organizational complexity beyond simple spherical assemblies, forming hierarchical supramolecular architectures with dendritic branching (1–3 μm branch widths). These patterns suggest directional polymerization influenced by the crystallographic orientations of the olivine substrate. Tubular networks (panel f) display continuous membrane-like morphologies that may function as primitive compartmentalization systems. Fibrillar assemblies exceeding 20 μm in length reflect extensive supramolecular organization mediated by hydrogen bonding and π–π stacking among phenylalanine residues, yielding mechanically robust polymeric structures. The emergence of these complex morphologies is driven by olivine dissolution under acidic conditions, which releases divalent metal cations (Mg^2+^ and Fe^2+^) and silicate species that act as both templates and cross-linkers. Crystal-like structures (panel k) indicate mineral-guided assembly along specific crystallographic planes, forming stable hybrid organic–inorganic architectures. Textured surfaces (panel i) reflect heterogeneous nucleation, where mineral interfaces organize structured proteinoid–substrate boundaries, potentially mirroring organic–inorganic coevolutionary pathways in which silicate minerals scaffolded early biochemical networks. Aggregate clusters (panel l, >25 μm) demonstrate higher-order hierarchical assembly in which microspheres and network components coalesce into multicomponent structures containing embedded spherical units and connective matrix elements. This suggests a staged formation pathway in which primary assemblies serve as modular building blocks. Mixed morphological features (panel j) further reveal dynamic assembly behavior responsive to local chemical gradients, enabling the simultaneous emergence of multiple structural motifs. Such plasticity highlights adaptive self-organization, wherein subtle variations in local chemistry, pH, or ionic strength redirect assembly pathways.

**Figure 3:**
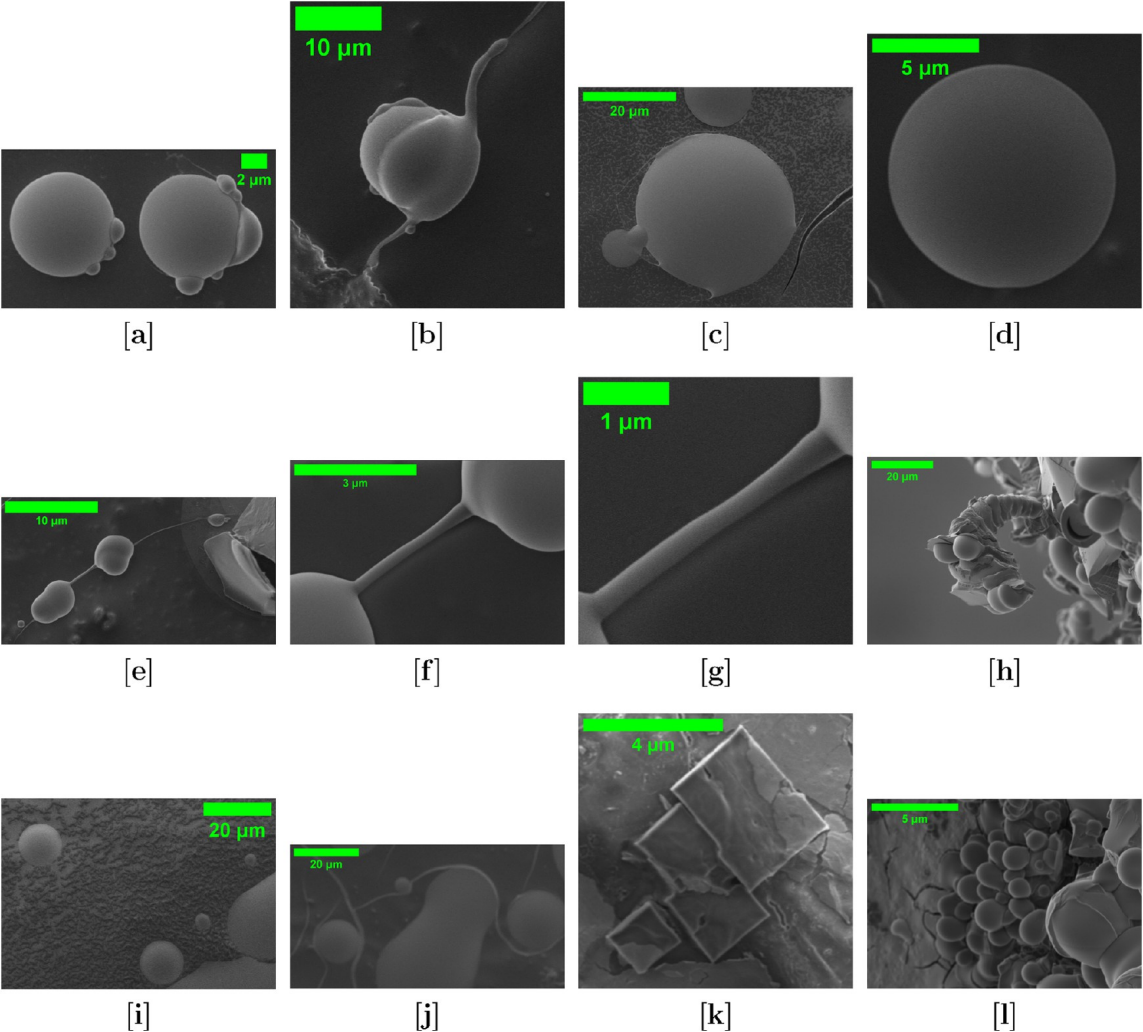
Scanning electron microscopy (SEM) images of glutamic acid:phenylalanine:aspartic acid (Glu:Phe:Asp) proteinoids in olivine acid solution reveal diverse self-assembly morphologies and cell-like features. **Top row [a–d]:** Spherical microspheres (2–15 μm) exhibit smooth surfaces and uniform shapes. Panel [a] shows budding-like pairs, suggesting reproduction. Panels [b–d] highlight isolated and colocated spheres with signs of coalescence or smooth surface boundaries. **Middle row [e–h]:** Advanced assemblies include dendritic branches (1–3 μm wide), tubular networks, and fibrils exceeding 20 μm. These suggest supramolecular organization and complex aggregation pathways. **Bottom row [i–l]:** Surface-textured spheres, mixed shapes, faceted crystalline-like structures, and large aggregates (>25 μm) appear. These may arise from proteinoid–mineral interactions, supporting both classical and novel assembly modes in olivine solution.

Proteinoid microspheres form through three thermodynamic phases: nucleation, growth, and maturation. This self-assembly process can be described using classical nucleation theory. Nucleation begins when the proteinoid polymer concentration exceeds the critical aggregation concentration (CAC), triggering spontaneous association driven primarily by hydrophobic interactions:

16 $${\mathrm{Proteinoid}}_{\mathrm{dissolved}}\overset{C>\mathrm{CAC}}{\to }\mathrm{Primary}\;\mathrm{nucleus}$$

The CAC depends on the balance between hydrophobic attraction and electrostatic repulsion and is governed by the free energy change Δ*G*_transfer_ associated with the transfer of proteinoid chains from the aqueous phase into a hydrophobic core. The height of the nucleation barrier determines the critical nucleus size according to classical nucleation theory,^173–175^ where the interfacial tension σ and the chemical potential difference Δ*μ* govern aggregation thermodynamics. Following nucleation, primary nuclei undergo rapid growth through monomer addition. Growth kinetics are consistent with diffusion-limited aggregation, in which the growth rate depends on surface area and local concentration gradients. The microsphere radius *R* increases as a function of the diffusion coefficient of proteinoid monomers *D*, the bulk monomer concentration *C*, and the equilibrium concentration *C*_eq_. Budding reproduction (Figure 4) exemplifies a form of primitive cellular behavior and occurs when microspheres reach a critical size, driven by surface tension minimization through Rayleigh–Plateau instability:

17 $${\mathrm{Microsphere}}_{\mathrm{parent}}\overset{\mathrm{budding}}{\to }{\mathrm{Microsphere}}_{\mathrm{parent}}^{*}+{\mathrm{Microsphere}}_{\mathrm{daughter}}$$

Budding is initiated when volume-dependent forces overcome surface energy barriers to deformation, a balance determined by surface tension, internal pressure, and microsphere volume. The complete assembly pathway proceeds through four sequential stages:

18 $$\mathrm{Stage}\;1:\;\mathrm{Glu}\text{−}\mathrm{Phe}\text{−}\mathrm{Asp}\;\mathrm{chains}\overset{\mathrm{hydrophobic}\mathrm{collapse}}{\to }\mathrm{Primary}\;\mathrm{nuclei}$$

19 $$\mathrm{Stage}\;2:\;\mathrm{Primary}\;\mathrm{nuclei}+\mathrm{monomers}\overset{\mathrm{growth}}{\to }\mathrm{Immature}\;\mathrm{microspheres}$$

20 $$\mathrm{Stage}\;3:\;\mathrm{Immature}\;\mathrm{microspheres}\overset{\mathrm{maturation}}{\to }\mathrm{Stable}\;\mathrm{microspheres}$$

21 $$\mathrm{Stage}\;4:\;\mathrm{Stable}\;\mathrm{microspheres}\overset{R>R_{\mathrm{critical}}\;}{\to }\mathrm{Budding}\;\mathrm{reproduction}$$

**Figure 4:**
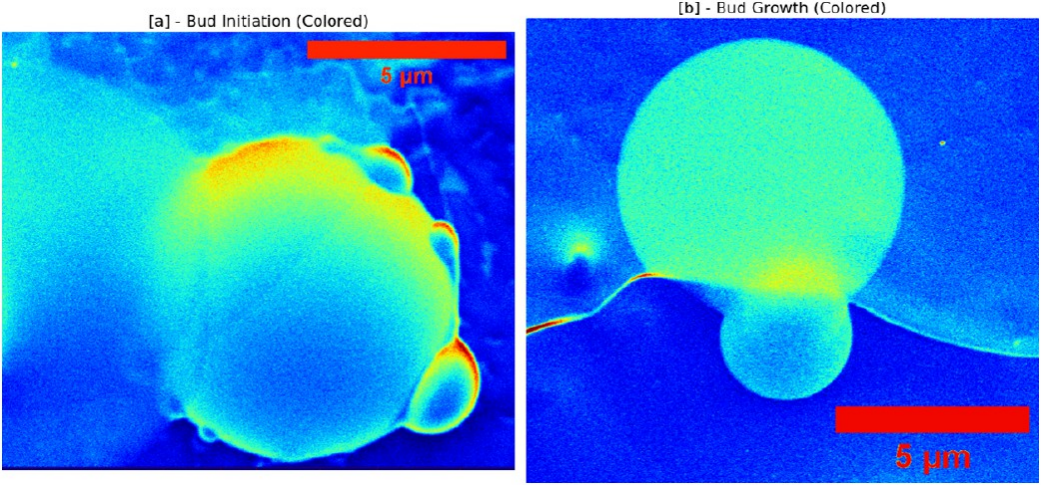
Proteinoid microsphere budding and reproduction in olivine acid solution reveal early features of cellular division. **Panel [a] – Bud initiation:** A mature parent microsphere (8 μm) exhibits multiple nascent buds at surface protrusions. False-color mapping shows high proteinoid density in the core (yellow–orange) and lower density at budding sites (blue–green). Budding occurs once the sphere exceeds the Rayleigh–Plateau critical radius, consistent with surface instabilities driven by pressure-mediated material redistribution. **Panel [b] – Bud growth:** A larger parent microsphere (12 μm) remains connected to a daughter sphere (4 μm) via a thin neck, yielding a 3:1 size ratio. The daughter sphere shows homogeneous density, indicating compartmentalization, while the neck suggests ongoing material transfer. Spherical morphologies reflect surface-tension minimization following budding.

Assembly is energetically favorable when the total free energy change satisfies Δ*G*_total_ < 0, reflecting the combined contributions of hydrophobic interactions, electrostatic effects, and surface free energy. The acidic olivine environment facilitates this process by providing optimal ionic strength, pH, and mineral surfaces that act as heterogeneous nucleation sites, thereby enhancing microsphere formation efficiency and reproducibility.

This figure shows a significant change (Figure S1, Supporting Information). It moves from simple proteinoid self-assembly to complex morphogenesis. This challenges how we usually think about abiotic structure development. Panel [a] shows the typical proteinoid microsphere shape. It has a perfect sphere with a smooth surface. This architecture reflects a balance in thermodynamics by minimizing surface tension. The uniform color from the yellow-green center to the blue edge shows a clear radial density gradient. Hydrophobic amino acids, like phenylalanine, gather in the middle. Meanwhile, hydrophilic groups, such as glutamic and aspartic acid, face the water. This initial state shows a stable setup. Here, the total free energy, Δ*G*_total_ = Δ*G*_hydrophobic_ + Δ*G*_electrostatic_ + Δ*G*_surface_, is at a local minimum. This forms a metastable structure. It can last a long time under steady conditions, but it can easily change if disturbed. Figure S1­[b] shows a major change. The microsphere develops in a new way. It creates a complex dendritic structure similar to how neurons look. Yellow-green linear channels appear around the central soma. This shows that high-density proteinoid conduits are forming. They can extend up to 8 μm from the main structure. This creates a network with branching patterns like a fractal. This morphogenesis seems driven by mechanical instabilities and chemical gradients. Local changes in pH, ionic strength, or mechanical stress disrupt the uniform spherical shape. This promotes growth in specific directions. The branching pattern shows a clear hierarchy. Primary branches lead to secondary and tertiary projections. This suggests a self-organizing process. It may be driven by diffusion-limited aggregation or reaction-diffusion mechanisms. These processes can create complex patterns from simple beginnings.

This neuron-like development probably happens through a phase change. It shifts from a uniform gel state to a mixed network structure. This change is driven by the local chemical environment or mechanical stress in the microsphere. The olivine acid solution creates a lively chemical environment. In this setting, the mineral matrix dissolves, releasing two types of cations: Mg^2+^ and Fe^2+^. These cations, along with silicate species, can act as cross-linking agents or chelators for the proteinoid polymers. When these species move into the microsphere interior, they form concentration gradients. These gradients can trigger local sol–gel transitions. This means some areas may gel while others stay fluid. Differential swelling and contraction create mechanical stress. This stress can cause fractures or channels. Over time, these fractures get stabilized and reinforced by more proteinoid deposition. This process leads to the formation of the dendritic structure we see. This process follows a reaction-diffusion equation (eq 22).

22 $$\frac{\partial C}{\partial t}=D\nabla^{2}C+R\left(C\right)$$

Here, *C* is the local proteinoid concentration, *D* stands for the diffusion coefficient, and *R*(*C*) explains the reaction kinetics that control gelation.

This neuron-like morphogenesis extends beyond visual resemblance, revealing structures that may support primitive information processing and signal transmission. Dendritic branches (Figure S1, panel b) create extensive surface area for environmental interaction, potentially enabling selective uptake of chemical signals or nutrients analogous to biological dendrites. The branching pattern establishes microsphere connectivity, forming networks capable of electrochemical or biochemical signal propagation. Unlike budding reproduction, this morphogenesis involves internal differentiation of the parent structure into specialized regions that function cooperatively within a larger network. Continuous connections between central soma and peripheral branches maintain system-wide coordination while enabling regional functional specialization-a pathway toward multicellular-like organization where individual proteinoid units assume distinct roles while remaining physically integrated. This observation illuminates nervous system origins: complex neural structures can emerge naturally from simple chemical systems under appropriate environmental conditions. The olivine acid environment provides chemical gradients and conditions conducive to this morphogenetic transformation, suggesting analogous processes may have occurred in prebiotic hydrothermal or mineral-rich settings where biological neural network precursors formed through abiotic processes. Pseudocolored SEM imaging (Figure 5, 20 μm scale) reveals detailed microsphere architecture. Jet colormap visualization highlights elongated branching connections between spherical bodies resembling dendrites, with smaller buds emerging from larger central spheres in dynamic growth patterns. Red intensity at connection bases suggests thicker, more developed nucleation sites forming neuron-like networks. Connection geometry shows nonrandom branching angles and lengths resembling synaptic architecture. Color gradients from blue to green to red along connections indicate material density or structural maturity transitions, with green regions representing intermediate growth phases. This supports the hypothesis that proteinoid systems recapitulate early neuronal development, with branching scaffolds enabling signal transmission or material exchange.

**Figure 5:**
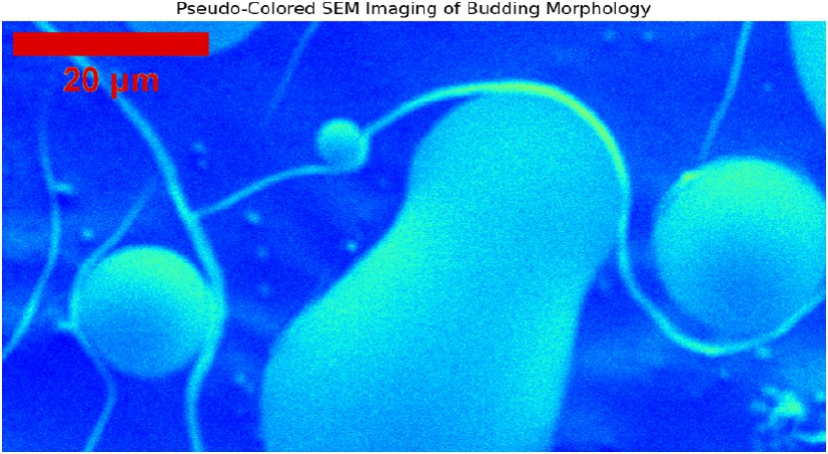
Pseudo-Colored SEM Imaging of Budding Morphology. This SEM image uses a jet colormap to show the detailed budding structures of proteinoid microspheres. The scale bar indicates 20 μm. The pseudocoloring shows changes in surface shape. It highlights possible neuron-like links between the round formations. The branching patterns and connections look like dendritic networks. This similarity needs more study to understand their role in proteinoid systems.

The term “neuron-like” refers to structural similarities with biological neurons. It describes features like soma-like central bodies, axon-like projections, and dendritic-like surfaces. However, it does not suggest that these structures function like biological neural cells. At the single-structure level (Figure 7), we see four unique neuron-like features. Panel (a) displays a spherical proteinoid microsphere (about 2–3 μm) with irregular, membrane-like protrusions. These extend from a smooth central core, resembling a neuronal soma with dendritic branches. Panel (b) shows a larger microsphere (about 8–10 μm). Its surface is heavily textured and looks like a cauliflower. It has many fine protrusions, similar to dendritic spines on pyramidal neurons. Panel (c) shows a smooth, cylindrical tube. Its diameter is about 0.5 to 1 μm, and it is longer than 10 μm. The tube has a uniform diameter and a consistent surface texture. It is similar in structure to a myelinated axon. Panel (d) shows three soma-like compartments (about 5–8 μm) linked by a stable tube. This structure resembles how neurons connect. The suppression of Rayleigh–Plateau instability indicates that the tube wall is reinforced by the polymer. At the network level (Figure 6), these morphological elements form a large, connected structure. It spans about 50 μm. Tubular conduits, measuring 0.5 to 2 μm in diameter, connect spherical nodes that range from 3 to 8 μm. These conduits branch through junctions and T-junctions, creating a network. The node degree distribution shows degrees of 4 to 5 at hub nodes. Its fractal structure, with a dimension of about 2.03 (see Table 1), aligns well with biological neural network patterns. Enclosed hollow compartments (5–15 μm) are visible. They show membrane-bounded areas like compartmentalized neural domains. All these structural features—soma-like bodies, axon-like projections, dendritic elaborations, multinode connectivity, and compartmentalization—form naturally from abiotic proteinoid self-assembly in the olivine acid environment, without any biological guidance. Their rise from basic amino acid chemistry in natural conditions shows that the principles behind neural-like shapes—like diffusion-limited aggregation, reaction-diffusion dynamics, and surface tension minimization—are universal, not just biological.

**Figure 6:**
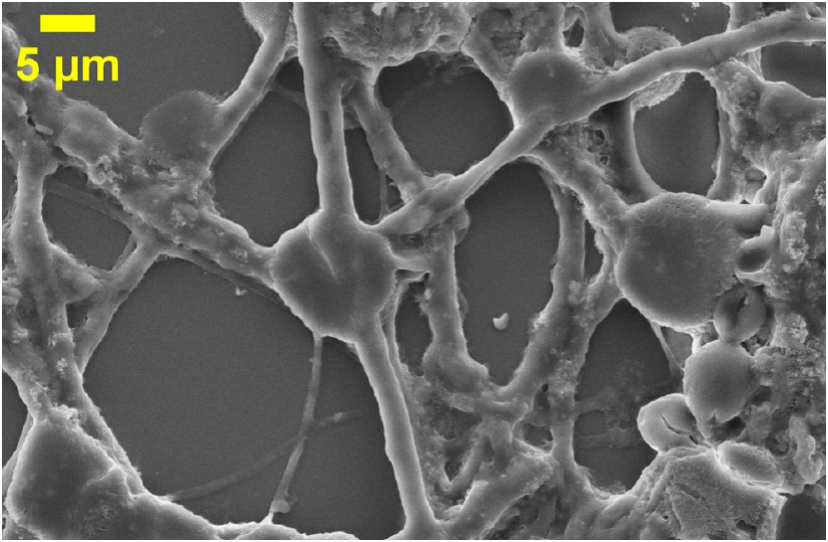
Scanning electron microscopy (SEM) image of a large-scale proteinoid neural network architecture (scale bar: 5 μm). The image reveals a spatially extended, interconnected structure spanning 50 μm, in which multiple morphological motifs coexist within a continuous network. Network topology: Tubular conduits (∼0.5–2 μm diameter) connect spherical node bodies (∼3–8 μm diameter), forming a multiply connected architecture. Node connectivity ranges from simple chains to hub-like junctions (degree 4–5), consistent with quantitative morphometric analysis and reminiscent of biological neural network organization. Soma-like nodes: Spherical bodies vary from isolated smooth microspheres to textured aggregates with surface protrusions. Some nodes exhibit submicron budding structures, indicating ongoing structural evolution within the network. Axon-like conduits: Smooth cylindrical tubes extend 10–25 μm between nodes, maintaining nearly constant diameter. Occasional branching and T-junctions enhance connectivity. The contrast between smooth tubes and textured nodes suggests distinct assembly mechanisms. Junction structures and compartments: Multiple conduits converge at compact junction bodies, forming branch-like geometries. Enclosed hollow compartments (5–15 μm) are also visible, indicating membrane-bounded regions integrated within the network. Fractal organization: The branching geometry is consistent with a fractal dimension *D*_f_ ≈ 2.03, reflecting scale-invariant, space-filling organization across micron scales. Overall, the image demonstrates that proteinoid systems can spontaneously generate interconnected, multiscale network architectures featuring nodes, conduits, branching junctions, and compartments, arising from abiotic self-assembly processes.

**Figure 7:**
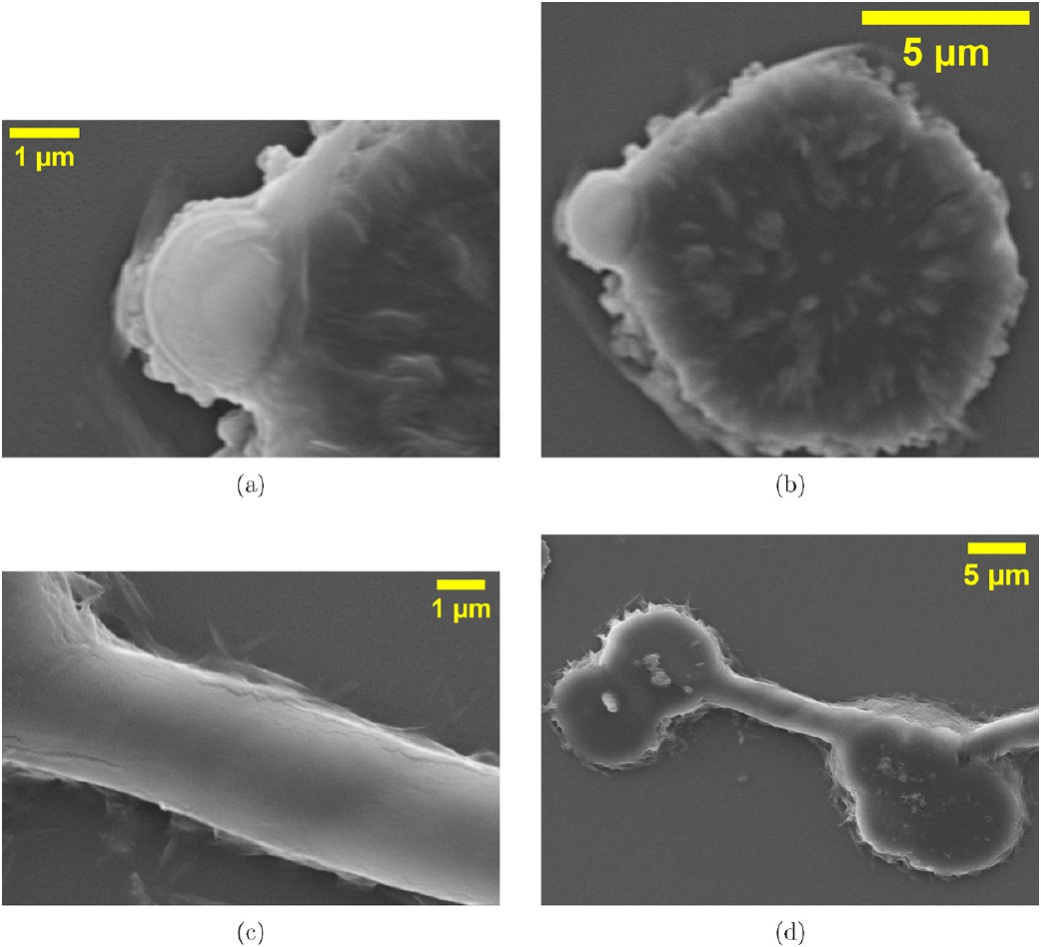
Scanning electron microscopy (SEM) images showing neuron-like morphologies in Glu:Phe:Asp proteinoid systems. The term neuron-like is used strictly in a morphological sense, referring to soma-like bodies, axon-like tubular projections, and dendritic-like surface features, without implying biological neural function. (a) Soma-like body with surface elaborations (scale bar: 1 μm): A spherical proteinoid microsphere (∼2–3 μm) with irregular membrane-like protrusions radiating from a smooth central core, analogous to a neuronal soma with dendritic extensions. (b) Textured soma with multiple protrusions (scale bar: 5 μm): A larger microsphere (∼8–10 μm) exhibiting a highly irregular, dendritic-like surface. (c) Axon-like tubular projection (scale bar: 1 μm): A smooth cylindrical tube (diameter ∼0.5–1 μm, length >10 μm) displaying uniform diameter and longitudinal texture, consistent with anisotropic growth along proteinoid-induced ionic gradients. (d) Interconnected multicompartment structure (scale bar: 5 μm): Three soma-like compartments (∼5–8 μm) linked by a stable tubular connection, forming a network reminiscent of interneuronal connectivity. Suppression of Rayleigh–Plateau instability suggests structural reinforcement of the connecting tube. Together, these morphologies demonstrate that soma-like compartments, axon-like projections, and network connectivity can emerge spontaneously from proteinoid self-assembly.

**Table 1:** Quantitative Morphometric Parameters Comparing Pure Proteinoid and Proteinoid-Olivine Structures^a^

Metric	Pure Proteinoid	Proteinoid-Olivine
Fractal Dimension (*D*_f_)	1.918	2.030
*R* ^2^ (fit quality)	0.9999	0.9999
Branch Points	1492	157
End points	490	26
Branching Ratio	3.04	6.04
Mean Node Degree (μ)	3.22	3.11
Lacunarity (Λ)	0.398	0.007

a Fractal dimensions were calculated using box-counting method with 12 box sizes spanning 2-4096 pixels. Branching metrics derived from skeletonized binary images. Lacunarity computed via gliding box algorithm across 25 box sizes. All measurements achieved exceptional fit quality (*R* ^2^ > 0.999), confirming scale-invariant fractal organization.

### Comparative Analysis of Dendritic Morphogenesis in Proteinoid and Physical Systems

#### Quantitative Morphometric Analysis of Proteinoid Networks

We performed fractal dimension analysis, branching metrics, node degree distribution, and lacunarity measurements on representative proteinoid structures (Figure 8, Table 1). Box-counting fractal dimension analysis reveals distinct morphological complexity between pure proteinoid and proteinoid-olivine systems. Pure proteinoid microspheres exhibit a fractal dimension of *D*_f_ = 1.918 (*R* ^2^ = 0.9999), indicating relatively smooth, regular boundaries characteristic of thermodynamically driven self-assembly. In contrast, proteinoid-olivine hybrids demonstrate elevated fractal complexity with *D*_f_ = 2.030 (*R* ^2^ = 0.9999), approaching the theoretical maximum of 2.0 for planar structures. This increased dimensionality suggests that mineral templating introduces surface roughness and hierarchical branching that extends the structure’s space-filling capacity beyond simple spherical geometry.

**Figure 8:**
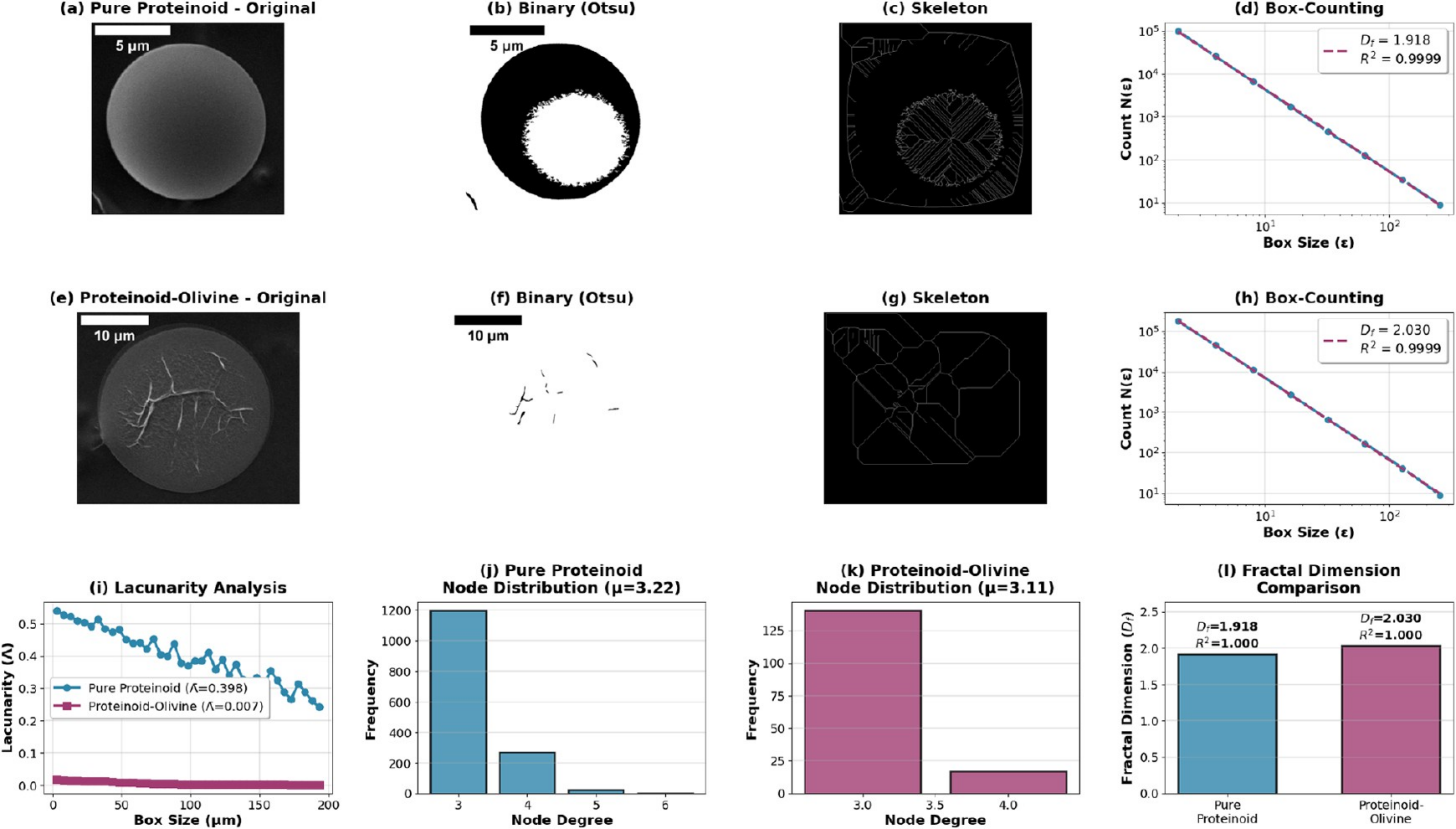
Quantitative morphometric analysis of proteinoid networks via box-counting fractal dimension, branching topology, and lacunarity measurements. Top row (a-d): Pure proteinoid microsphere analysis. (a) SEM image showing smooth spherical morphology with surface texture (scale bar: 5 μm). (b) Binary segmentation via Otsu thresholding reveals boundary complexity. (c) Skeletonization extracts network topology, identifying 1492 branch points and 490 end points. (d) Box-counting analysis yields *D*_f_ = 1.918 (*R* ^2^ = 0.9999), consistent with regular boundary structure. Middle row (e–h): Proteinoid-olivine hybrid analysis. (e) SEM image displays dendritic branching architecture (scale bar: 10 μm). (f) Binary image captures fragmented network structure. (g) Skeleton reveals compact, highly connected topology with 157 branch points serving 26 end points. (h) Elevated fractal dimension *D*_f_ = 2.030 (*R* ^2^ = 0.9999) indicates increased space-filling complexity. Bottom row (i–l): Comparative metrics. (i) Lacunarity analysis shows pure proteinoids exhibit heterogeneous space-filling (Λ = 0.398) while olivine-templated structures display near-uniform distribution (Λ = 0.007). (j, k) Node degree histograms confirm ternary branching dominance (peak at degree 3) in both systems, with mean values of μ = 3.22 and μ = 3.11 respectively. (l) Summary comparison highlights 5.8% increase in fractal dimension upon mineral templating, positioning proteinoid-olivine networks within the biological neural arbor range (*D*_f_ = 1.9–2.2). The exceptional fit quality (*R* ^2^ > 0.999) validates the box-counting methodology and confirms scale-invariant fractal organization across 2–3 orders of magnitude in spatial scale.

The measured fractal dimensions align with biological neural arbors (*D*_f_ = 1.9–2.2 for cortical pyramidal neurons) and exceed those of purely physical dendrites formed via electrodeposition (*D*_f_ = 1.6–1.8). This position in the biological range shows that even abiotic chemistry can lead to neural-like structures. Key physical principles, such as diffusion-limited aggregation and reaction-diffusion, can form these structures in mineral-organic systems.

Branching analysis via skeletonization quantifies network topology (Table 1). Pure proteinoids contain 16,656 total branch pixels with 1492 branch points and 490 end points, yielding a branching ratio of 3.04. The mean node degree of 3.22 indicates predominantly ternary branching. Proteinoid-olivine systems show fewer total branch pixels (6831) but a dramatically higher branching ratio of 6.04, with 157 branch points serving only 26 end points. This higher ratio shows denser, more connected networks. They have better connectivity for their size. This setup boosts how efficiently information spreads in limited spaces.

Node degree distributions provide insight into network architecture. Both systems exhibit peak frequencies at degree 3, confirming ternary bifurcation as the dominant branching motif. Pure proteinoids show secondary populations at degree 4, indicating occasional quaternary junctions. The mean node degree stays consistent−μ = 3.22 for pure and μ = 3.11 for olivine-templated. This shows that local branching rules follow basic physical laws. These likely include surface tension minimization and mechanical stress distribution. Mineral substrates can change the overall shape, but they do not alter these rules.

Lacunarity analysis quantifies spatial heterogeneity, with lower values indicating more homogeneous space-filling. Pure proteinoids exhibit mean lacunarity Λ = 0.398 (range: 0.243–0.541), reflecting the irregular boundary structure evident in panel (c) of Figure 8. Proteinoid-olivine systems show dramatically reduced lacunarity (Λ = 0.007, range: 0.003–0.020), approaching the theoretical minimum for uniform structures. This near-zero lacunarity shows that mineral templating creates orderly, even dendritic networks. This is good for efficient signal propagation in computational designs.

Proteinoid-olivine systems are well-suited for distributed information processing. This is due to their high fractal dimension (*D*_f_ = 2.030), a branching ratio of 6.04, a stable ternary node topology (μ = 3.11), and low lacunarity (Λ = 0.007). These numbers show that mineral-templated proteinoid assembly mirrors important shapes found in biological neural networks. This happens through simple physical self-organization. It supports the idea that mineral-organic interfaces in early life could help create proto-cognitive systems.

Neuron-like structures observed in proteinoid-olivine systems (Figure 9) exhibit morphological characteristics consistent with diffusion-limited aggregation (DLA) and reaction-diffusion processes. In purely abiotic systems such as metal electrodeposition or crystallization in supercooled liquids, dendritic patterns emerge from interfacial instabilities governed by local concentration gradients. The proteinoid-olivine hybrid system extends this physical mechanism through mineral-mediated chemical templating. Olivine dissolution releases divalent cations (Mg^2+^, Fe^2+^) and silicate species that establish localized ionic and pH gradients, disrupting the thermodynamic preference for spherical symmetry that dominates pure proteinoid assembly. These gradients direct polymerization along preferred crystallographic orientations and mechanical stress lines, transforming isotropic microspheres into anisotropic dendritic networks.

**Figure 9:**
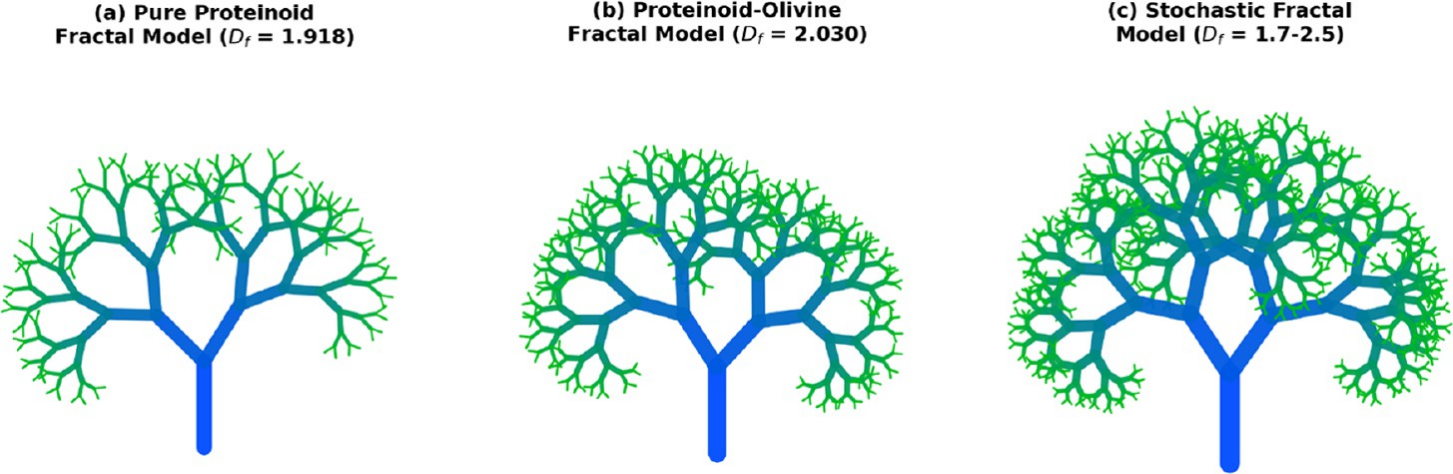
Stochastic fractal models simulating proteinoid dendritic morphogenesis with varying complexity levels. (a) Pure Proteinoid Fractal Model (*D*_f_ = 1.918): Simpler branching architecture with depth = 8 recursion levels, representing the relatively regular boundary structures observed in thermodynamically driven pure proteinoid self-assembly. The reduced branching complexity reflects minimal mineral templating influence, consistent with the measured fractal dimension of 1.918 from box-counting analysis. (b) Proteinoid-Olivine Fractal Model (*D*_f_ = 2.030): Intermediate complexity with depth = 9 recursion, capturing the enhanced space-filling capacity induced by olivine mineral templating. The elevated fractal dimension (2.030) approaches the theoretical planar maximum, indicating hierarchical branching driven by localized chemical gradients from olivine dissolution. (c) Stochastic Fractal Model (*D*_f_ = 1.7–2.5): Maximum complexity demonstration with depth = 10 recursion, showcasing the full range of biologically relevant fractal dimensions. The model incorporates stochastic perturbations in branching angles (±12°) and length decay (0.68–0.82) to emulate diffusion-limited aggregation and reaction-diffusion mechanisms observed in mineral-templated proteinoid assembly. The color gradient changes from dark blue in the center to light green at the edges. This mirrors the structure, going from the core to the branching dendrites. These models show that basic physical principles—like self-organized criticality, reaction-diffusion dynamics, and surface tension minimization—can create neural-like structures. They do this using simple recursive branching rules. This supports the idea that proto-cognitive network shapes formed naturally in early mineral-organic systems.^56,108,112^

Quantitative morphometric analysis confirms fractal organization in these dendritic arbors. The measured fractal dimensions (*D*_f_ = 1.918 for pure proteinoid, *D*_f_ = 2.030 for proteinoid-olivine; Table 1) fall within the range observed for biological neural dendrites (*D*_f_ = 1.9–2.2) and exceed values typical of purely physical dendrites (*D*_f_ = 1.6–1.8). Branching density follows a power-law relationship *N*_branches_ ∝ *L* ^*D*_f_^, characteristic of scale-free networks exhibiting self-organized criticality. This statistical framework, which governs pattern formation in diverse systems from snowflake crystallization to neuronal arborization, emerges spontaneously in proteinoid-olivine assemblies without external direction.

Unlike abiotic dendrites driven by single dominant gradients (thermal, electrical, or concentration), proteinoid morphogenesis operates within a multidimensional energy landscape. Hydrophobic collapse of phenylalanine residues, electrostatic repulsion between acidic amino acid side chains (glutamate, aspartate), and metal-carboxylate coordination chemistry collectively define a “soft matter” growth mechanism. This complexity enables the system to maintain structural robustness while retaining morphological plasticity—permitting formation of stable soma-like central bodies connected to branching conduits capable of directional signal transmission.

The transition from uniform spherical morphology to networked dendritic architecture represents a nonequilibrium phase transition driven by internal sol–gel dynamics. Classical physical dendritic growth (e.g., solidification fronts, electrochemical deposition) typically proceeds irreversibly under external control parameters such as temperature or applied voltage. In contrast, proteinoid branching exhibits adaptive responsiveness to local perturbations in pH, ionic strength, and mechanical stress at the mineral-organic interface. This environmental sensitivity enables regionalized differentiation within the parent structure, facilitating integration into larger interconnected networks—a developmental strategy reminiscent of early multicellular organization.

These findings demonstrate that fundamental physical instabilities governing dendritic growth—predating biological evolution—can spontaneously generate architectures functionally analogous to neural networks when operating in chemically complex mineral-organic systems. The quantitative correspondence between proteinoid fractal dimensions and biological neural arbors suggests that principles of optimal information distribution through branched networks represent universal physical constraints rather than exclusively biological innovations.^11,19^

### Temporal Dynamics and Regression Modeling of Electrochemical Impedance

The Nyquist plot (Figure 10a) illustrates how impedance evolves in the complex plane over time. Each color-coded trajectory corresponds to a distinct measurement cycle recorded at various time intervals. The plot forms a curved arc that dips downward, characteristic of nonideal capacitive behavior in the olivine–proteinoid system. This pattern likely arises from distributed capacitance or surface heterogeneities. As time progresses (from dark blue at *t* = 0 s to yellow at *t* ≈ 20,000 s), the arc initially expands, indicating an increase in charge-transfer resistance. It later contracts slightly, suggesting stabilization or onset of degradation processes. These temporal changes reflect evolving electrical properties, possibly due to ion migration or interfacial reactions under constant current conditions.

**Figure 10:**
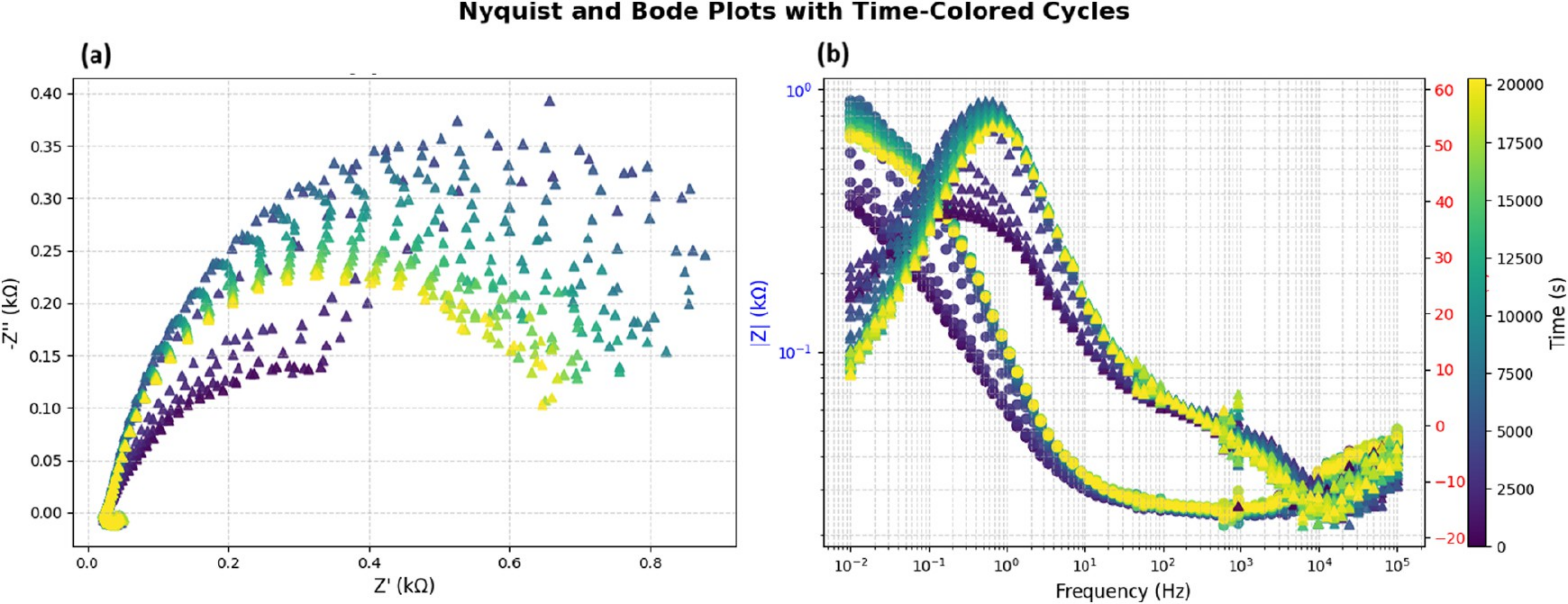
(a) Nyquist (left) and Bode (right) plots show galvanostatic impedance data for olivine-proteinoid samples. The points are colored by measurement time (s). The Nyquist plot shows real (Z′) versus imaginary (−*Z*″) impedance in kΩ, revealing temporal arc evolution. (b) The Bode plot shows magnitude *|Z|* (circles) and phase (triangles) against log frequency (Hz). It highlights both frequency and time dependencies.

The Bode plot (Figure 10b) complements the Nyquist analysis by showing frequency-dependent impedance magnitude (|*Z*|) and phase angle. As frequency increases, the impedance magnitude decreases—a typical behavior for RC circuits. The phase angle begins near 0° at high frequencies, indicating resistive dominance, and shifts to more negative values at lower frequencies due to increasing capacitive influence. Color-coded time progression reveals that early cycles (cooler tones) exhibit lower |*Z*| at low frequencies. This value peaks midexperiment and slightly decreases thereafter, echoing the trend seen in the Nyquist plot. This progression suggests an initial buildup of resistive contributions, followed by partial relaxation, possibly reflecting electrochemical adaptation in the proteinoid–olivine matrix.

EIS analysis of the olivine-proteinoid system shows complex changes over time. We can understand these dynamics by closely examining the impedance parameters. The mean impedance magnitude |*Z*| shows a three-phase evolution over the 20,267-s experiment, as seen in Table S2. The impedance magnitude can be expressed as eq 23:

23 $$\left|Z\right|=\sqrt{{\left(Z\right)}^{\prime 2}+{\left(Z\right)}^{\prime \prime 2}}$$

where *Z*′ represents the real component and *Z*″ represents the imaginary component of the complex impedance. In the initial phase (0–2500 s), the resistance rises from 0.072 to 0.082 kΩ. This change suggests that the system is stabilizing and that initial interactions are happening between the olivine surface and the proteinoid molecules.

The quick rise in impedance seen in Figure 11a from 2500 to 6000 s marks the most active part of the experiment. Here, |*Z*| peaks at 0.162 kΩ during cycle 9 (*t* = 6291 s), as shown in Table S2. This dramatic increase can be attributed to protein adsorption and surface modification processes that significantly alter the interfacial properties. The phase angle behavior in Figure 11b peaks at about 17° during this time. This shows improved capacitive behavior. The relationship between impedance magnitude and phase angle is governed by eq 24:

24 $$\phi =\arctan\left(\frac{Z^{\prime \prime }}{Z^{\prime }}\right)$$

where ϕ represents the phase angle. The rise in both |Z| and ϕ hints at a proteinoid layer forming. This layer serves as a dielectric barrier and boosts the system’s capacitance. The stabilization phase occurs from 6000 to 20,000 s. During this time, both the table data and Figure 11 show a steady drop in impedance magnitude to 0.140 kΩ. Meanwhile, the phase angle levels off at about 14°. This behavior shows that structural changes or partial desorption happen at the mineral-proteinoid interface. The standard deviation values in Table S2 show a similar pattern over time. Maximum variability, at 0.245 kΩ, happens during the peak impedance phase. This suggests varied surface interactions. The impedance response during this phase can be modeled using equivalent circuit parameters as shown in eq 25:

25 $$Z_{\mathrm{total}}=R_{s}+\frac{R_{\mathrm{ct}}}{1+j\omega R_{\mathrm{ct}}C_{\mathrm{dl}}}$$

**Figure 11:**
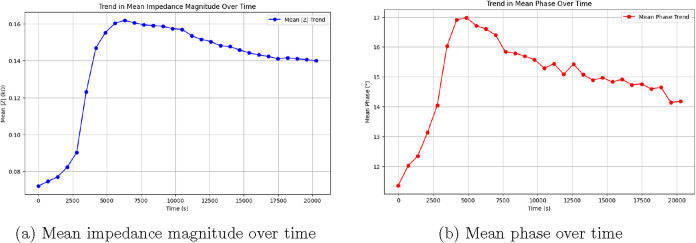
Electrochemical impedance spectroscopy (EIS) of the olivine–proteinoid system over a 20,000-s period. (a) The mean impedance magnitude |*Z*| exhibits a three-phase evolution: a gradual increase from 0.072 to 0.082 kΩ during the first 2500 s, a rapid rise to a peak of 0.162 kΩ at 6000 s, and a subsequent decrease to 0.140 kΩ by the end of the experiment. This behavior suggests initial equilibration, followed by surface modification or proteinoid attachment, and eventual stabilization. (b) The mean phase angle shows complementary dynamics, increasing from 11.4 to 17.0° before decreasing in two stages to 14.2°. These trends reflect evolving capacitive and resistive properties, consistent with changes in the protein layer and mineral–organic interface relevant to prebiotic surface processes.

Here, *R*_s_ stands for solution resistance. *R*_ct_ is the charge transfer resistance. *C*_dl_ is the double layer capacitance. ω represents angular frequency, and *j* is the imaginary unit. Analyzing the real and imaginary parts of impedance gives more insights into the system’s electrical features. Table S2 shows that the highest real impedance component *Z*′ hits 0.877 kΩ at cycle 9. At the same time, the maximum negative imaginary component – *Z*″ peaks at 0.394 kΩ. The ratio of these components decides if the system behaves more like a resistor or a capacitor. The impedance magnitude connects to the frequency response through eq 26:

26 $$Z\left(\omega \right)=Z^{\prime }-jZ^{\prime \prime }=\left|Z\right|e^{-j\phi }$$

This relationship shows how changes over time, shown in Figure 11, impact the electrical properties of the olivine-proteinoid interface. The drop in the *Z*″/*Z*′ ratio means a shift from mostly capacitive to more resistive behavior.

To implement Boolean logic gates using impedance data, we first threshold the mean impedance magnitude |*Z*| to binary values. For instance, values ≥ 0.14 kΩ are mapped to 1 (logic high), and values below this threshold to 0 (logic low). This binarization transforms continuous electrochemical measurements into digital signals suitable for logic gate operations.

Pairing inputs—such as consecutive binary values (e.g., *A* as current and *B* as previous via shift­())—sets up the gate evaluation framework, as shown in eq 27:

27 $$\mathrm{Binary}\;\mathrm{Input}=\{\begin{array}{ll} 1 & \mathrm{if}\;\left|Z\right|\geq 0.14\\ 0 & \mathrm{otherwise} \end{array}$$

The AND gate outputs 1 only if both inputs are 1, simulating logical conjunction, as shown in eq 28.

28 AND(a,b)=a∧b

When applied row-wise to the DataFrame, this gate identifies scenarios where high impedance in consecutive cycles indicates sustained “active” electrochemical states.

The OR gate outputs 1 if at least one input is 1, representing logical disjunction, as shown in eq 29.

29 OR(a,b)=a∨b

This gate captures transient or alternating high-impedance events across cycles.

The XOR gate outputs 1 if the two inputs differ, representing logical exclusive or, as shown in eq 30.

30 XOR(a,b)=a⊕b=(a∧¬b)∨(¬a∧b)

This is particularly useful for detecting phase transitions between states of low and high resistance.

The NAND and NOR gates are the negations of AND and OR, respectively, while the NOT gate inverts a single input. Their logic definitions are given in eq 31.

31 NAND(a,b)=¬(a∧b),NOR(a,b)=¬(a∨b),NOT(a)=¬a

These inverse logic operations help characterize electrochemical deactivation and reversals in impedance behavior. The temporal evolution of impedance-based Boolean logic operations reveals distinct phases in the proteinoid material’s electrical behavior (Figure 12 and Table S3). Initially, from *t* = 0 s to *t* = 4195.7 s, the system maintains a consistent low-impedance state with mean |*Z*| values ranging from 0.072 to 0.123 kΩ, all below the 0.14 kΩ threshold. During this phase, the binary input remains at 0, resulting in predictable logic outputs: AND operations yield 0 (requiring both inputs to be high), OR operations produce 0 (since both consecutive inputs are low), and XOR outputs remain at 0 (no state transitions detected). The inverted gates (NAND, NOR, and NOT) consistently output 1, reflecting the system’s stable low-resistance configuration.

**Figure 12:**
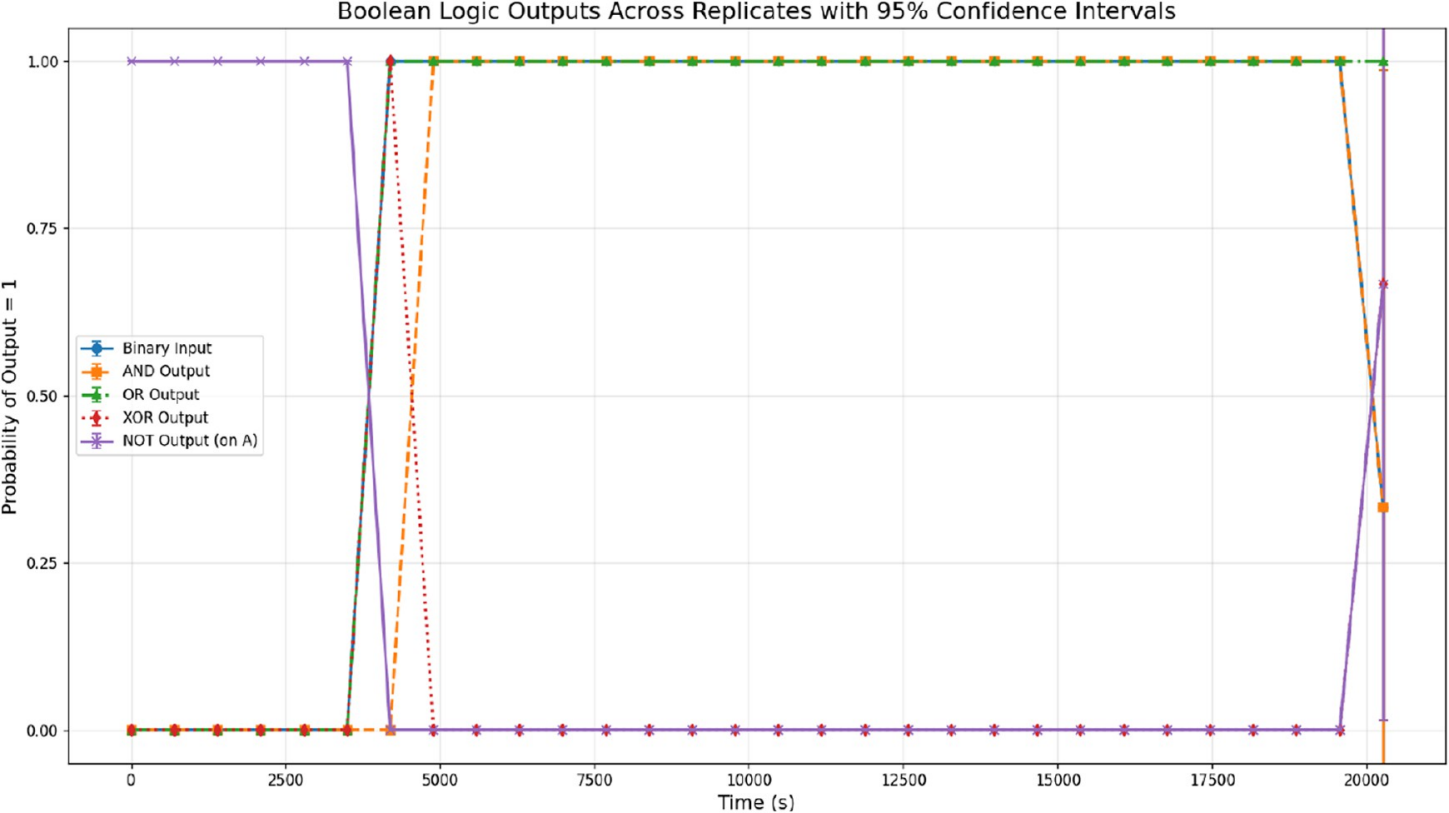
Boolean logic gates applied to binary-thresholded impedance data derived from the mean impedance magnitude |*Z*| obtained from galvanostatic impedance spectroscopy of olivine–proteinoid samples. The impedance signal is converted into binary states using a threshold of 0.14 kΩ, where values ≥ 0.14 kΩ are assigned logic 1 (high resistance) and values below the threshold are assigned logic 0 (low resistance). Inputs *A* and *B* correspond to consecutive binary states (current and previous time point, respectively), enabling temporal logic operations based on the evolving impedance dynamics. The binary input sequence is shown as blue circles, while selected gate outputs are displayed as follows: AND (green squares, dashed), OR (orange triangles, dash-dotted), XOR (red diamonds, dotted), and NOT applied to *A* (purple ×, solid). Error bars represent 95% confidence intervals calculated across replicate measurements. NAND and NOR gates are omitted for clarity. The system remains in a logic-low state until *t* ≈ 4196 s, followed by a prolonged logic-high phase until *t* ≈ 19568 s corresponding to the impedance plateau. During this interval, the AND output remains high, suggesting sustained activation or memory-like behavior within the electrochemical response. XOR peaks mark transition points at the onset and termination of the high state, indicating switching events in the thresholded signal. These observations demonstrate that the temporal impedance dynamics of proteinoid–mineral systems can generate logic-like patterns when interpreted through binary thresholding, supporting their potential as unconventional computing substrates linking analog electrochemical processes with digital representations.

A critical transition occurs at *t* = 4195.7 s when the impedance crosses the threshold to 0.146934 kΩ, triggering the first high binary state. This moment marks a significant shift in logic patterns, with the XOR gate detecting the state change by outputting 1, while the OR gate switches to 1 as at least one input becomes high. The AND gate remains at 0 during this transition since only the current input *A* is high while the previous input *B* remains 0. This transition point demonstrates the system’s sensitivity to electrochemical changes and its potential for edge detection in computing applications.

The most remarkable feature emerges during the extended high-impedance plateau from *t* = 4894.3 s to *t* = 19568.1 s, where impedance values stabilize between 0.140 and 0.161 kΩ. Throughout this 14673-s period, both consecutive inputs *A* and *B* maintain high states, resulting in sustained AND outputs of 1 and OR outputs of 1, while XOR outputs remain at 0 (indicating no state transitions). This prolonged activation period suggests the material’s capacity for stable memory retention, with the impedance plateau potentially representing a metastable electrochemical state that could encode information over extended timeframes.

The final transition at *t* = 20267.0 s reveals the system’s return to a low-impedance state (0.139957 kΩ), creating another XOR spike as the state change is detected. At this point, input *A* drops to 0 while input *B* remains at the previous high state, causing the AND gate to deactivate while the OR gate maintains its high output. This asymmetric transition pattern differs from the initial threshold crossing, suggesting that the material’s electrochemical response exhibits hysteresis or path-dependent behavior that could be exploited for more complex computational tasks.

The inverted logic operations (NAND, NOR, NOT) provide complementary perspectives on the system’s behavior, consistently reflecting the inverse of their positive counterparts throughout all phases. The NOT gate’s output pattern particularly emphasizes periods of low electrical activity, while NAND and NOR gates highlight deactivation phases. These comprehensive logic mappings demonstrate that proteinoid materials can simultaneously perform multiple Boolean operations, with their natural impedance fluctuations serving as the computational substrate. The clear temporal correlation between electrochemical states and digital logic outputs suggests these materials could function as bioinspired computing elements, potentially enabling reservoir computing architectures where the material’s intrinsic dynamics perform computational tasks without requiring explicit programming of logic circuits.

We chose 0.14 kΩ as the binarization threshold based on a statistical analysis. This analysis examined the data distribution and the trends observed over time in the galvanostatic impedance spectroscopy measurements. The overall mean of |*Z*| across all 30 cycles is approximately 0.143 kΩ, with a median of 0.147 kΩ, indicating a slight skew toward higher values during the plateau phase. The time-series plot shows a clear change: for the first six cycles (*t* = 0 to *t* = 3497.4 s), values remain below 0.14 kΩ. This low-resistance state likely results from early charging or limited interfacial buildup. Then, starting at *t* = 4195.7 s, values rise above this level, indicating higher resistance.

Using 0.14 kΩ—just below the median—clearly separates these regimes. It captures the rise to the peak value of approximately 0.162 kΩ at *t* = 6291.5 s, followed by a mild decline. This choice also helps reduce false positives in binary classification. The threshold aligns with the data’s natural breakpoint, as confirmed by a histogram showing a bimodal tendency with clusters below 0.12 kΩ and above 0.14 kΩ.

The threshold was refined to improve logical interpretation for Boolean gate operations. This adjustment allows digital switching to better match the electrochemical dynamics in the olivine-proteinoid system. A lower threshold, such as 0.12 kΩ, might wrongly label midrise values as high, confusing transient buildup with stable high-impedance states. Conversely, a higher threshold like 0.15 kΩ could miss some plateau segments, undercounting sustained activation periods essential for gates like AND, which require consecutive high inputs.

At 0.14 kΩ, the binary inputs show clear patterns. From cycles 6 to 28, a long sequence of binary 1s creates steady AND outputs. The XOR gate marks exact transitions at the beginning and end, while the NOT gate inverts to highlight low-resistance phases. This threshold balances sensitivity to data variability—with a standard deviation of about 0.05 kΩ—and robustness. This is crucial for bioinspired computing, where impedance changes mimic neural thresholding mechanisms.

### Distribution Analysis of Oscillation Parameters in Proteinoid Systems

The statistics show a very varied oscillatory system (Table 2). There’s clear variability in all the measured parameters. The amplitude distribution shows a strong right skew (skewness = 5.79) and very high kurtosis (52.88). This means small-scale fluctuations dominate the system, with rare but significant burst events. The system shows “burst-dominated” dynamics. Its mean amplitude is 2.12 mV, but it can peak at 50.56 mV. Most oscillations stay below 2.53 mV, which is the 75th percentile. Yet, rare high-amplitude events cause significant changes in the overall signal variance. This statistical signature hints at complex random processes. These processes have heavy-tailed distributions, not simple Gaussian noise.

**Table 2:** Descriptive Statistics of Amplitudes, Periods, and Frequencies Derived from Galvanostatic Measurements of the Olivine–glu_phe_asp Proteinoid System^a^

index	count	mean	std	min	25%	50% (median)	75%	max	skewness	kurtosis
Amplitude (mV)	509.0	2.1222	4.0006	0.1004	0.2335	0.6002	2.5259	50.5600	5.7911	52.8811
Period (s)	510.0	176.3608	206.2950	5.0000	33.2500	87.0000	251.2500	1528.0000	2.0099	5.4659
Frequency (Hz)	510.0	0.0217	0.0262	0.0007	0.0040	0.0115	0.0301	0.2000	2.3735	7.6698

a A total of 511 peaks were detected, yielding 509 amplitudes (peak-to-trough, mV) and 510 periods (s); frequencies were computed as the inverse of periods (Hz). Amplitudes show high variability, with a mean of 2.12 mV and a standard deviation of 4.00 mV, indicating intermittent bursts superimposed on smaller fluctuations, likely arising from heterogeneous electrochemical processes. Periods average 176 s with substantial dispersion (206 s), suggesting quasi-periodic behavior rather than regular oscillations. Frequencies cluster around 0.022 Hz, while FFT analysis reveals a dominant DC component (0 Hz). Overall, the statistics reflect complex, burst-like dynamics relevant to bio-inspired electrochemical oscillator modeling.

The period analysis shows a complex time structure. The average interpeak interval is 176.36 s, but there is a lot of variability. The standard deviation is 206.30 s. The period distribution ranges from 5 to 1528 s. This shows that the system works across different time scales at the same time. The moderate right-skew (2.01) and high kurtosis (5.47) show that there are quick oscillatory phases mixed with longer quiet periods. This temporal variation shows that the median period of 87 s is much shorter than the mean. This suggests that the system often has quick oscillations. Yet, it also experiences longer pauses of low activity. These patterns are typical of systems with memory effects or state-dependent dynamics.

The frequency analysis shows that low frequencies dominate the system. The average frequency is 0.022 Hz, which means cycles last about 46 s. The frequency distribution shows similar traits to the period data. It has a skewness of 2.37 and a kurtosis of 7.67. This confirms that multiple time scale dynamics are present. Identifying 0 Hz as the main FFT component shows a strong DC bias. This suggests there might be baseline drift or slow processes affecting the faster dynamics. This frequency signature matches diffusion-driven or thermal processes in bioinspired materials. In these materials, molecular transport happens naturally on these time scales.

The signal changes over time (Figures 13 and 14). It starts with strong activity, then gradually decreases and stabilizes. The strong 60 mV burst at *t* = 5000 s, followed by a drop to under 5 mV by *t* = 80,000 s, shows a system relaxing from an excited state. This evolution pattern shows energy dissipation mechanisms at work. It suggests that the proteinoid system needs outside help or energy to keep a steady oscillation. The background-removed analysis separates the oscillatory component from the drift. This reveals 511 detectable peaks. These peaks form the statistical basis for the next analysis.

**Figure 13:**
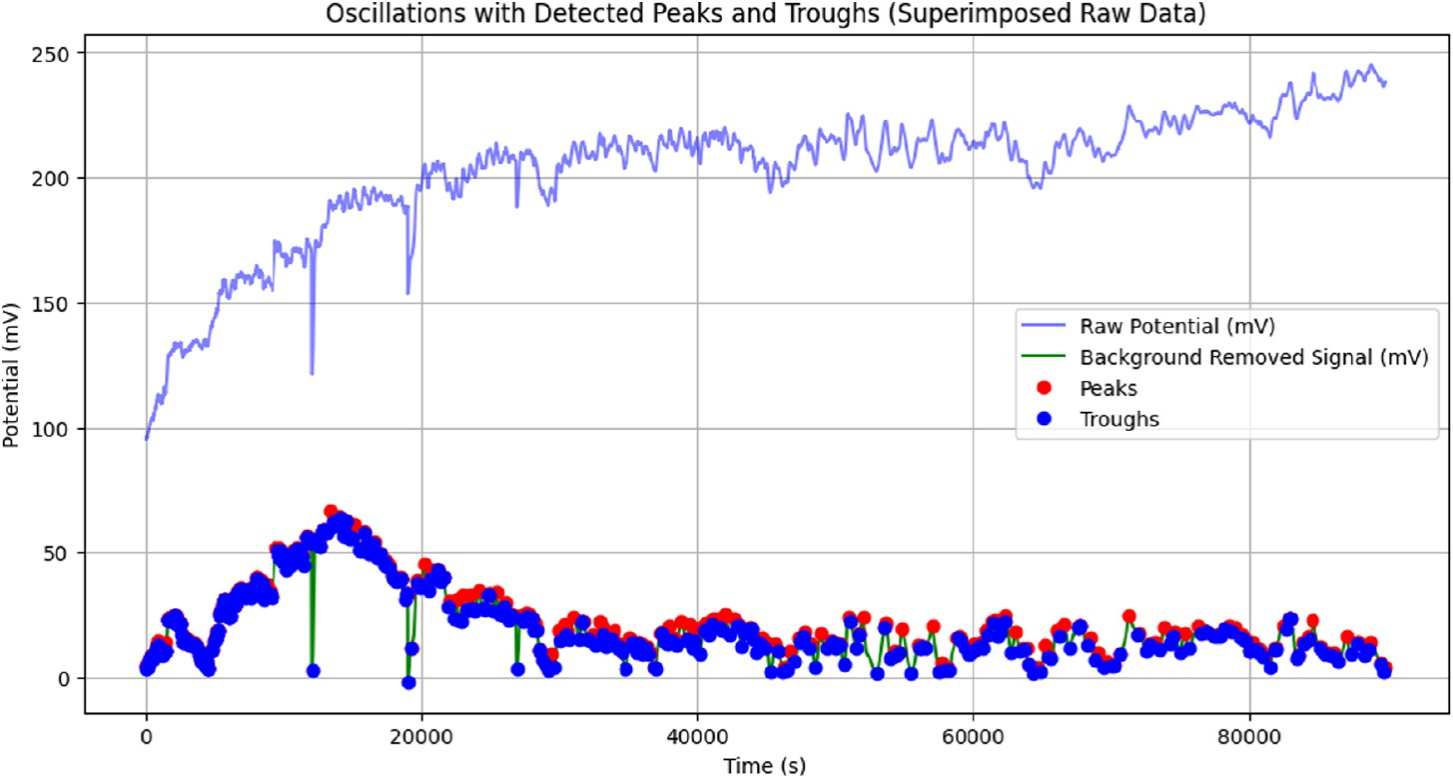
Spontaneous potential oscillations (mV) in the olivine–glu_phe_asp proteinoid system over 93,000 s. Raw data (blue) shows a gradual upward drift, likely due to baseline or environmental effects, while the baseline-subtracted signal (green) isolates the oscillatory component. Detected peaks (red circles) and troughs (blue circles) identify 511 peaks, yielding 509 amplitudes and 510 interpeak periods. A pronounced burst of 60 mV at *t* ≈ 5000 s suggests rapid electrochemical activation or charge accumulation, followed by progressive damping to sub-5 mV fluctuations by *t* ≈ 80,000 s, indicating relaxation or stabilization. This behavior is consistent with the statistical analysis, which shows predominantly small oscillations punctuated by rare large events, quasi-periodic timing, and low-frequency dominance. Overall, the data reveal burst-driven, dynamically evolving electrical activity, highlighting the self-organizing electrochemical properties of proteinoids relevant to unconventional computing and biomimetic sensing.

**Figure 14:**
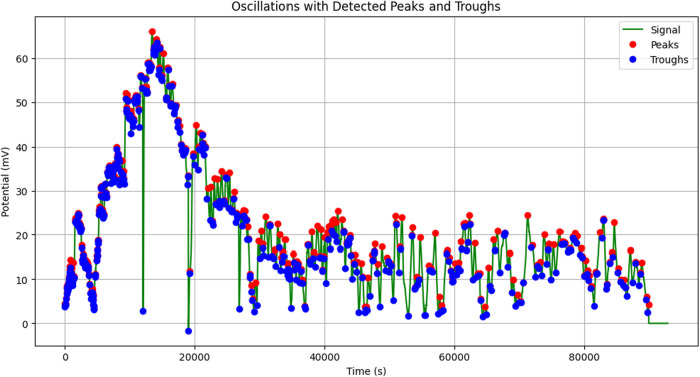
Natural oscillations in the baseline-subtracted potential (mV) of the olivine–glu_phe_asp proteinoid system over ∼93,000 s. Peaks (red circles) and troughs (blue circles) are marked on the processed signal (green). A strong burst of 60 mV occurs at *t* ≈ 5000 s, followed by a rapid decay into smaller, irregular fluctuations that gradually diminish by *t* ≈ 80,000 s, indicating an initial activation phase followed by quasi-periodic self-organizing dynamics. A total of 511 peaks were detected. The mean amplitude is 2.12 mV (SD 4.00 mV; max 50.56 mV), reflecting burst-dominated variability. The mean interpeak period is 176 s (SD 206 s; range 5–1528 s), corresponding to a mean frequency of 0.022 Hz.

Statistical testing clearly rejects both exponential and Poisson models for the oscillatory behavior (Figures 15 and 16). The *p*-values are highly significant: 6.32 × 10^–11^ for the Kolmogorov–Smirnov test, and effectively zero for the chi-squared tests. Exponential fitting failed, indicating that interevent intervals are not memoryless. This suggests the presence of correlations, feedback mechanisms, or deterministic factors influencing the dynamics.

**Figure 15:**
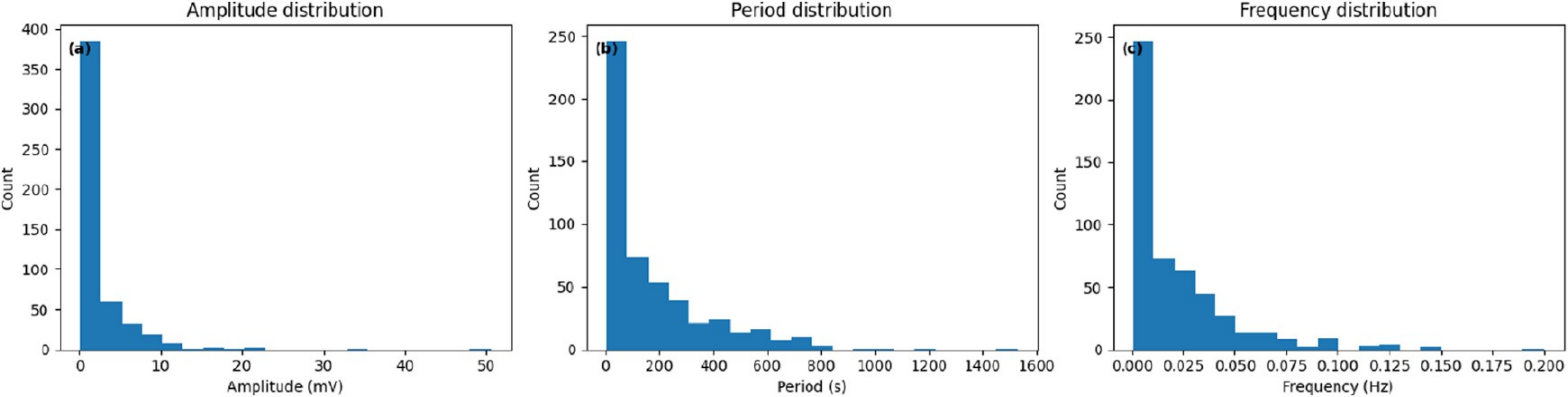
Histograms of amplitudes (mV), periods (s), and frequencies (Hz) for spontaneous oscillations in the olivine–glu_phe_asp proteinoid system following background removal. (a) The amplitude distribution (509 events) is strongly right-skewed (skewness ≈ 5.79; kurtosis ≈ 52.88), with most values below 2.53 mV and rare bursts up to 50.56 mV. The mean amplitude is 2.12 mV (SD 4.00 mV), indicating predominantly low-intensity oscillations punctuated by high-magnitude events. (b) Periods (510 intervals) show moderate skewness (≈2.01) with a mean of 176 s (SD 206 s; range 5–1528 s), consistent with quasi-periodic dynamics. (c) Frequencies cluster at low values (mean 0.022 Hz; range 0.00065–0.2 Hz), reflecting slow, diffusion-driven processes. Overall, the distributions highlight irregular, burst-like, and nonstationary behavior relevant to bioinspired electrochemical oscillators. Although frequency is defined as the inverse of the period on an event-by-event basis (*f* = 1/*T*), the period and frequency histograms represent independent distributions; their similar shapes arise from nonlinear transformation and binning effects rather than from a violation of this inverse relationship.

**Figure 16:**
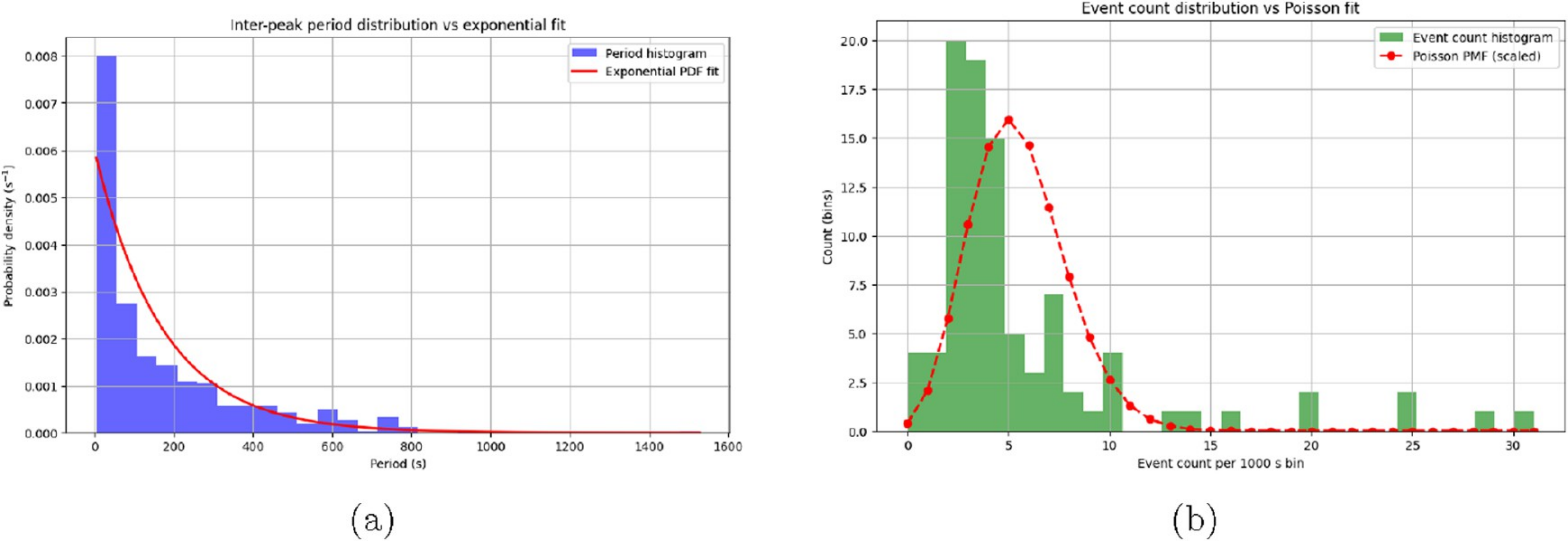
Histograms of oscillation periods and event counts derived from spontaneous potential fluctuations in the olivine–glu_phe_asp proteinoid system, compared with Poisson-based models. (a) A density histogram of 510 interpeak periods (blue) is shown with an overlaid exponential PDF (red; loc = 5.0, scale = 171.36 s, λ ≈ 0.0058 Hz). Although the distribution is right-skewed, it deviates strongly from the exponential model, as confirmed by a Kolmogorov–Smirnov test (statistic = 0.153, *p* = 6.32 × 10^–11^), indicating nonmemoryless, correlated dynamics. (b) A histogram of peak counts per 2.0 s bin (green) is compared with a Poisson PMF (red; λ ≈ 0.01). A chi-squared test (χ^2^ = 0.07, *p* = 0.0) rejects the Poisson fit, revealing overdispersion and event clustering. Increasing the bin size to ∼182 s (mean period) does not restore Poisson behavior (χ^2^ = 2.56 × 10^11^, *p* = 0.0). Overall, the results demonstrate strongly non-Poissonian oscillations, suggesting correlated or deterministic processes that may be exploitable for bioinspired and unconventional computing models.

The overdispersion observed in the peak count analysis—where the variance exceeds the mean—implies event clustering rather than purely random occurrence. Such non-Poissonian behavior is characteristic of systems with feedback loops, autocatalytic reactions, or external environmental influences. These elements introduce temporal dependencies and connections between events.

This proteinoid system has complex patterns. It shows heavy-tailed distributions, multiscale timing, and nonrandom event clustering. These features hint at deep dynamics that could be used for unique computing applications. The burst-like behavior, with its rare high-amplitude events, looks like neural spikes. The quasi-periodic nature, which has variable cycle lengths, may support how we process time. Simple stochastic models have failed. This shows that these systems are complex enough for advanced computational tasks. Also, the call for better filtering techniques highlights key methods for future research. These findings show that proteinoid systems are good options for bioinspired sensing and neuromorphic computing. Their irregular and adaptive dynamics may be better than traditional periodic oscillators.

### Thermodynamic Limits and the Landauer Principle in Proteinoid–Olivine Oscillations

The Landauer Principle establishes a fundamental thermodynamic limit for information processing, stating that the erasure of one bit of information requires a minimum energy expenditure:^176–178^

32 $$E_{L}=k_{B}T\;\ln2$$

where *k*_B_ = 1.381 × 10^–23^ J/K is the Boltzmann constant and *T* is the absolute temperature. At our experimental temperature of 25 °C (298.15 K), this theoretical limit is *E*_L_ = 2.853 × 10^–21^ J per bit. This limit represents the minimum energetic cost imposed by the second law of thermodynamics for any irreversible computational operation involving information erasure.

Our galvanostatic measurements revealed spontaneous electrical oscillations with 511 detected peaks over approximately 93,000 s (Figure 17). The amplitude distribution showed mean 2.122 mV (standard deviation: 4.00 mV, range: 0.10–50.56 mV), with pronounced heavy-tailed characteristics (skewness = 5.79, kurtosis = 52.88). The mean interpeak period was 176.361 s, corresponding to an average oscillation frequency of approximately 0.0057 Hz. Each detected peak represents a discrete informational event analogous to neural spiking, where the system’s non-Poissonian statistics (confirmed by Kolmogorov–Smirnov test, *p* < 6.32 × 10^–11^) suggest memory-dependent processes rather than random noise.

**Figure 17:**
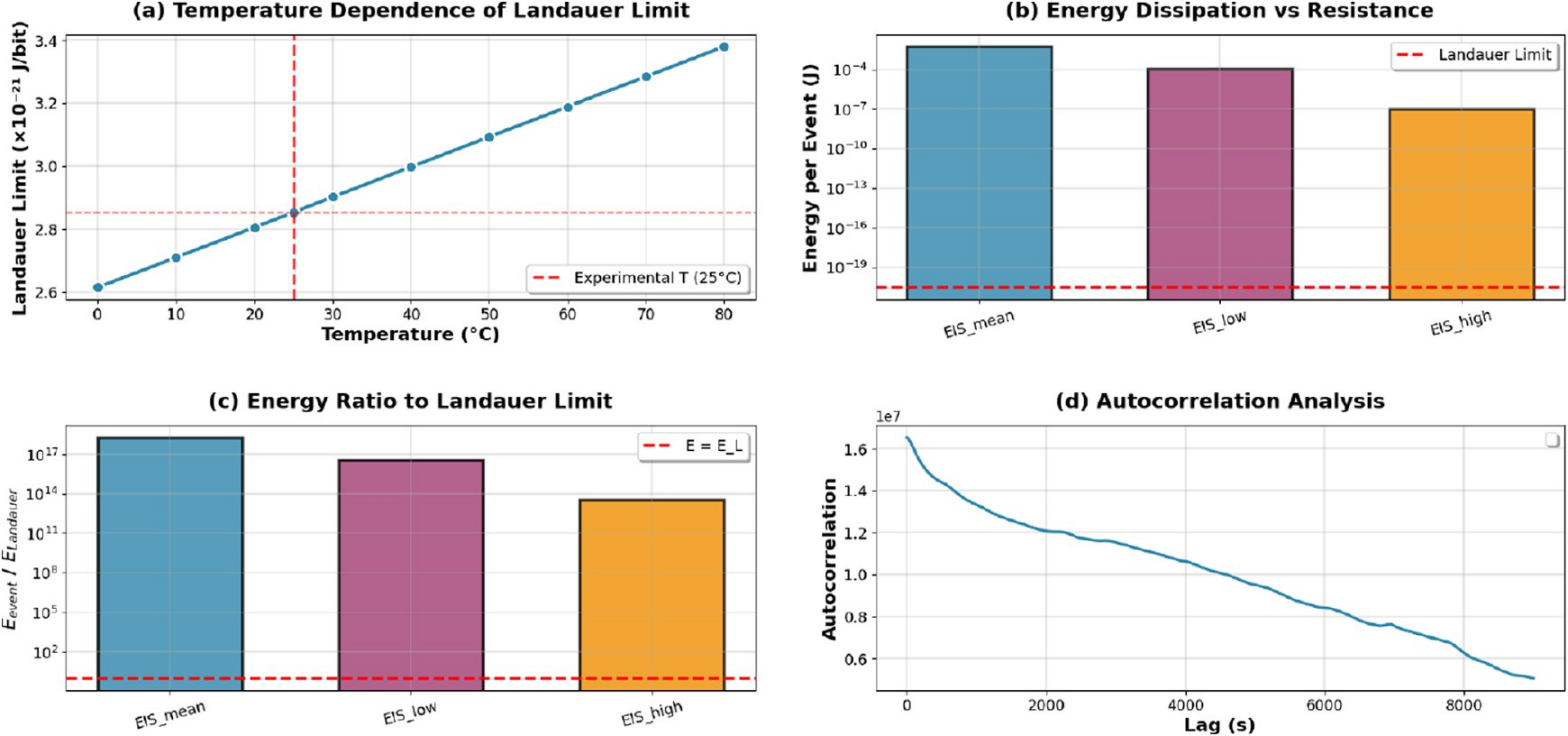
Landauer Principle analysis of proteinoid–olivine oscillatory dynamics. (a) Temperature dependence of Landauer limit: Linear relationship *E*_L_ = *k*_B_ *T *ln 2 across 0–80 °C range (blue line with markers). Red dashed lines indicate experimental temperature (25 °C) and corresponding *E*_L_ = 2.853 × 10^–21^ J/bit. The modest 29% increase over 80 °C demonstrates weak temperature sensitivity of fundamental thermodynamic constraints. (b) Energy dissipation vs resistance regime: Energy per event calculated for three representative resistance values from EIS measurements. Blue bar (EIS_mean, R = 18.88 Ω): *E*_event_ = 5.39 × 10^–3^ J. Purple bar (EIS_low, R = 1 kΩ): *E*_event_ = 1.02 × 10^–4^ J. Orange bar (EIS_high, R = 1 MΩ): *E*_event_ = 1.02 × 10^–7^ J. Red dashed line shows Landauer limit. Logarithmic scale reveals 6 orders of magnitude variation in energy dissipation across impedance regimes. (c) Energy ratio to Landauer limit: Normalized comparison showing system operates 10^13^–10^18^ times above thermodynamic minimum depending on resistance. Even in most efficient regime (high impedance), energy exceeds *E*_L_ by 13 orders of magnitude, consistent with biological neural computation (∼10^11^ × *E*_L_). Red dashed line at unity indicates hypothetical Landauer-limited operation. (d) Autocorrelation analysis: Temporal correlation structure of oscillatory signal over 9,000-s lag range. Slow exponential decay indicates long-range memory effects and persistent correlations incompatible with memoryless Poisson processes. Characteristic decay time scale τ ∼ 3000 s exceeds mean interevent interval (176 s) by a factor of 17, confirming history-dependent dynamics. Analysis based on 511 detected peaks over 93,000-s measurement duration, mean amplitude 2.122 mV, mean period 176.361 s.

To quantify energy dissipation per event and compare to the Landauer limit, we modeled the system’s instantaneous power dissipation as

33 $$P\left(t\right)=\frac{V{\left(t\right)}^{2}}{R_{\mathrm{eff}}}$$

where *V*(*t*) is the measured potential and *R*_eff_ is the effective resistance at the proteinoid–olivine interface. The total energy dissipated over the measurement duration is

34 $$E_{\mathrm{total}}=\int_{0}^{T}P\left(t\right)dt=\int_{0}^{T}\frac{V{\left(t\right)}^{2}}{R_{\mathrm{eff}}}dt$$

Electrochemical impedance spectroscopy (EIS) measurements revealed resistance values spanning 6 orders of magnitude depending on measurement conditions and frequency (Table 3). Stable galvanostatic time scans at 1000 Hz yielded mean impedance |*Z*| = 18.88 Ω (standard deviation: 0.39 Ω), while frequency-dependent EIS showed values ranging from ∼1 kΩ to >1 MΩ. This variability reflects the complex, frequency-dependent, and state-dependent electrical properties of the hybrid mineral–organic interface.

**Table 3:** Energy Dissipation Analysis Comparing Measured Oscillatory Events to the Landauer Limit across Three Resistance Regimes from Electrochemical Impedance Spectroscopy^a^

Resistance Regime	*R*(Ω)	*E*_total_ (J)	*E*_event_ (J)	*E*_event_/*E*_*L*_
EIS mean (time scan)	18.88	2.756	5.39 × 10^–3^	1.89 × 10^18^
EIS low (intermediate)	1.0 × 10^3^	5.20 × 10^–2^	1.02 × 10^–4^	3.57 × 10^16^
EIS high (capacitive)	1.0 × 10^6^	5.20 × 10^–5^	1.02 × 10^–7^	3.57 × 10^13^
*Landauer limit at 25* °*C*: *E*_*L*_ = 2.853 × 10^–21^ *J*/*bit*				
*Measurement conditions: 511 peaks, 93,000 s duration, mean amplitude 2.122 mV, mean period 176.361 s*				

a Energy per event calculated by integrating power dissipation *P*(*t*) = *V*(*t*)^2^/*R* over 93,000-second measurement period and dividing by 511 detected peaks. All configurations exceed Landauer limit by >10^13^, demonstrating the fundamental gap between real computation and the thermodynamic minimum.

Using these measured resistance values, we calculated energy dissipation per oscillatory event across three representative regimes (Figure 17b,c; Table 3). At the low-resistance limit (*R* = 18.88 Ω, corresponding to galvanostatic time-scan conditions), total energy dissipation over the measurement period was *E*_total_ = 2.76 J, yielding energy per event *E*_event_ ≈ 5.39 × 10^–3^ J. This exceeds the Landauer limit by a factor of ∼1.89 × 10^18^—18 orders of magnitude above the fundamental thermodynamic minimum.

At intermediate resistance (*R* = 1 kΩ, typical of EIS measurements), *E*_total_ = 0.052 J and *E*_event_ ≈ 1.02 × 10^–4^ J, exceeding *E*_*L*_ by ∼3.57 × 10^16^. At high resistance (*R* = 1 MΩ, representing capacitive-dominated regimes), *E*_total_ = 5.2 × 10^–5^ J and *E*_event_ ≈ 1.02 × 10^–7^ J, still ∼3.57 × 10^13^ times the Landauer limit. Even in the most electrically efficient scenario—where impedance reaches mega-ohm values—the system operates 13 orders of magnitude above the fundamental thermodynamic minimum. This is consistent with biological computation, where neural spike generation requires ∼10^9^ ATP molecules (∼10^–10^ J per spike), approximately 10^11^ times *E*_L_.

The Landauer limit exhibits linear temperature dependence: *E*_L_(*T*) = *k*_B_ *T* ln 2 (Figure 17a). Across the biologically relevant temperature range (0–80 °C), *E*_L_ increases from 2.62 × 10^–21^ J/bit to 3.38 × 10^–21^ J/bit—only a 29% increase over an 80-degree span. At physiological temperatures (37 °C, 310 K), *E*_L_ = 2.97 × 10^–21^ J/bit, representing merely a 4% increase over our experimental conditions. This weak temperature sensitivity (∂*E*_L_/∂*T* = *k*_B_ ln 2 = 9.57 × 10^–24^ J/(K·bit)) suggests that fundamental thermodynamic constraints on information processing remain relatively constant across biologically relevant temperature ranges.

However, the proteinoid–olivine system’s oscillatory dynamics show strong empirical temperature dependence through altered reaction kinetics, ion mobility, proton transfer rates, and phase transition thermodynamics—effects that dominate over the modest shift in Landauer constraints. The autocorrelation analysis (Figure 17d) reveals long-range temporal correlations with characteristic decay time scales exceeding 8000 s, indicating persistent memory effects incompatible with simple Markovian dynamics. The exponential decay at long lags suggests that while oscillations are quasi-periodic, they retain information about prior states over time scales far exceeding individual event durations.

These findings illuminate several key principles: Real physical systems performing computation necessarily operate far above the Landauer limit due to finite-time processing constraints, dissipative dynamics, and the need for error correction. The 10^13^–10^18^ gap observed here quantifies this fundamental inefficiency. The energy cost for each informational event decreases as system resistance increases. This shows that impedance engineering is key for energy-efficient bioinspired computing. The energy dissipation varies by 6 orders of magnitude, from 10^–7^ to 10^–3^ J per event. This shows how crucial interfacial electrochemistry is for computational efficiency. Non-Poissonian burst statistics and heavy-tailed amplitude distributions show that events carrying information come from collective, correlated processes. These processes display self-organized criticality instead of just random thermal fluctuations. This is characteristic of complex adaptive systems operating near phase transitions. Temperature changes in proteinoid-mineral systems might have led to early thermal sensing. This helped organisms adapt to their environment. Such adaptations provided advantages before genetic regulatory systems developed.

The large energy surplus compared to *E*_L_ does not break thermodynamic rules. Instead, it shows practical limits. Biological and bioinspired systems focus on speed, strength, and parallel processing rather than thermodynamic efficiency. The proteinoid–olivine system operates in the fast-oscillation regime with a mean period of ∼176 s. It functions far from quasi-static equilibrium. This necessitates substantial energy dissipation to maintain stable, repeatable informational states. Future work should look into how changing mineral composition or pH can help lower energy use. This should also keep the system’s computational abilities intact.

### Electrochemical Conditioning and Steady-State Behavior in Proteinoid-Based Cyclic Voltammetry

Cyclic voltammetry reveals a clear *activation phase*, characterized by strong initial current responses that rapidly transition to a more stable, steady-state regime. The first cycle exhibits the highest electrochemical activity observed in the series, with cathodic currents reaching a peak of −1553.37 μA at −0.5 V, and anodic currents peaking at 811.89 μA. This pronounced initial response represents the most intense electrochemical behavior recorded across all 100 cycles. Such strong early activity is visible both in the overlay visualization of the voltammograms (Figures 18 and 19) and in the quantitative changes observed in the statistical analysis (Table S1). This behavior is likely due to critical surface activation phenomena, including the penetration of electrolyte into the proteinoid matrix, the formation of electrochemical double layers, and the utilization of accessible redox-active species. The statistical metrics further emphasize this phase: the highest absolute values for current and area under the curve (AUC) are concentrated in the initial cycles. This underscores the importance of the activation period in shaping the long-term electrochemical characteristics of the system. The transition from the initial activation phase to a steady-state regime occurs over approximately 15–20 cycles. This shift is evident in the color gradient of the cyclic voltammetry (CV) overlay and the exponential decay trends observed in parameter tracking plots. During the conditioning phase (cycles 2–15), the minimum cathodic currents decrease dramatically from −857 μA to approximately −40 μA, while the maximum anodic currents fall from 297 μA to the range of 50–60 μA. This represents more than a 10-fold reduction in electrochemical activity. The area under the curve (AUC) exhibits distinctive behavior during this phase. It begins with a high positive value of 178.85 μA·V in cycle 1, briefly dips into the negative at cycle 4 with a minimum of – 20.53 μA·V, and subsequently stabilizes around 5–8 μA·V for the remaining cycles. The presence of a transient negative AUC suggests a temporary reversal in net charge transfer direction during conditioning. This may reflect the depletion of surface-bound redox-active species and the onset of reversible redox processes, which are likely to influence the system’s long-term electrochemical behavior.

**Figure 18:**
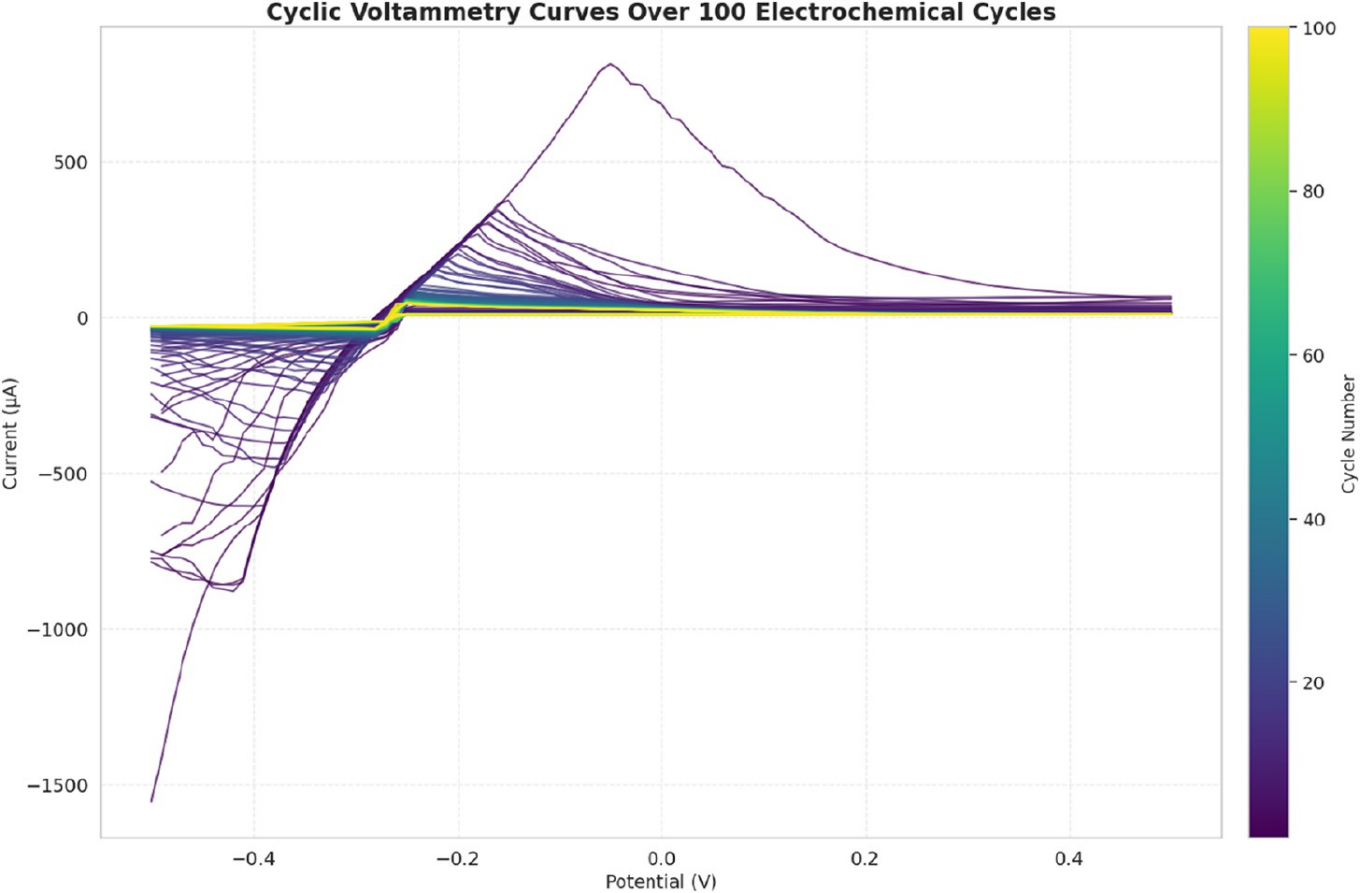
Overlay of 100 consecutive cyclic voltammetry (CV) curves showing the electrochemical conditioning of a proteinoid-based electrode system. The color gradient from purple (early cycles) to yellow (later cycles) illustrates the transition from an initially dynamic response to a stable steady state. The first cycle exhibits strong electrochemical activity, with cathodic currents reaching −1553 μA at −0.5 V and anodic currents peaking at 812 μA near −0.1 V, consistent with surface activation, electrolyte penetration, and irreversible redox processes. Cycles 2–15 show rapid conditioning, with current magnitudes decreasing by over an order of magnitude and voltammogram shapes converging toward quasi-reversible behavior. From cycles 20–100, the CV profiles are highly reproducible, with symmetric anodic and cathodic features centered around −0.25 V and currents confined within ±50 μA, indicating stable, diffusion-controlled redox processes. The absence of anomalies in later cycles confirms successful conditioning within the aqueous window (±0.5 V). These results suggest that 15–20 preconditioning cycles are required for stable operation, supporting the suitability of proteinoid-based electrodes for sensing, energy storage, and bioinspired computing applications.

**Figure 19:**
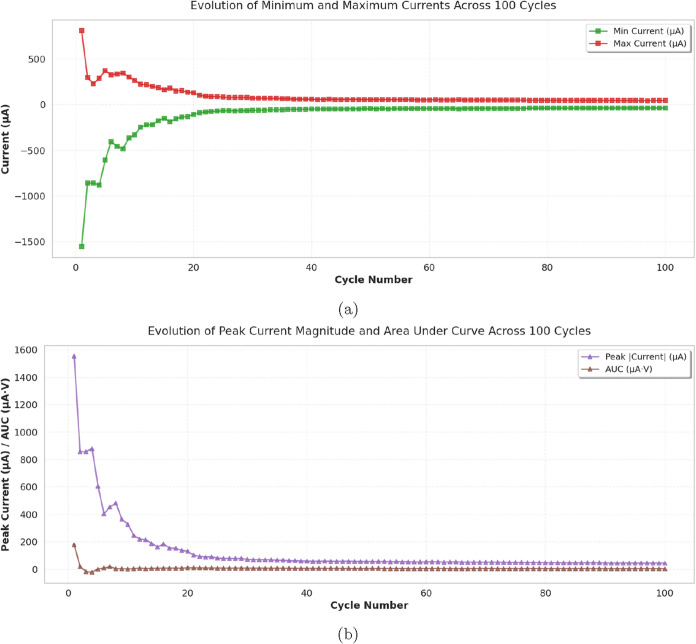
Evolution of electrochemical parameters over 100 cyclic voltammetry cycles in proteinoid-based electrodes. (a) Minimum (green) and maximum (red) currents show typical conditioning behavior. Cycle 1 exhibits strong activation (−1553.37 μA minimum; 811.89 μA maximum), followed by rapid stabilization within cycles 2–15. Thereafter, minimum currents converge near −40 μA and maximum currents near 50–60 μA, indicating electrochemical equilibration and stable redox behavior. (b) Peak absolute current (purple) and area under the curve (AUC; brown) decrease sharply after the first cycle. Peak current declines from 1553.37 to 50 μA, while AUC drops from 178.85 μA·V to stable values of 5–8 μA·V after a brief negative transient in cycle 4. Statistical dispersion is highest during early cycles, underscoring the transition from initial activation to a reproducible steady-state electrochemical regime.

The detailed statistical analysis reveals a diverse electrochemical response during the conditioning process. The initial values are extreme and heavily influence the overall parameter distributions. In contrast, the steady-state behavior is confined to significantly narrower ranges. The minimum current values exhibit high variability, with a standard deviation of 222.18 μA—a reflection of the pronounced cathodic activity in the first cycle. In comparison, maximum current values show less dispersion, with a standard deviation of 107.13 μA and a mean value of 99.66 μA. These trends are further supported by the statistics of the peak absolute current, confirming the consistency and reliability of the measurement approach. The area under the curve (AUC) offers additional insight into the electrochemical evolution. It spans a wide range from −20.53 to 178.85 μA·V, with a mean value of 8.17 μA·V. This range captures both the intense charge transfer during the activation phase and the stabilized redox behavior associated with the conditioned electrode surface.

The observed conditioning behavior has significant implications for the practical deployment of proteinoid-based electrochemical devices. It establishes clear protocols for electrode preparation and defines the expected evolution of bioinspired electrochemical systems. Specifically, device initialization should incorporate 15–20 conditioning cycles to ensure stable and reproducible performance. Moreover, the long-term stability observed from cycles 20 to 100 demonstrates that such systems are viable for applications in sensing, energy storage, and neuromorphic computing. The electrochemical memory manifested during the conditioning phase highlights the transition from a dynamic to a stable operating regime. This behavior may prove valuable for adaptive or learning-enabled electrochemical platforms. The narrow steady-state current window of approximately ±50 μA reflects excellent electrochemical stability, indicating minimal electrode degradation and strong long-term durability. These properties position proteinoid-based systems as promising candidates for the development of sustainable, bioinspired electrochemical technologies that combine biologically informed design with robust electronic performance.

The progressive decrease in peak current observed during repeated cyclic voltammetry cycles likely reflects interfacial conditioning of the proteinoid–mineral system. Processes such as surface passivation, partial depletion of reactive ionic species, or stabilization of the electrochemical double layer can reduce the effective charge-transfer activity during early cycles. After this initial conditioning phase, the system approaches a quasi-steady electrochemical regime in which the CV profiles vary only gradually between cycles. This stabilized state provides a reproducible electrochemical environment suitable for subsequent impedance measurements and analysis of the system dynamics.

### Optimization of Pulse Amplitude in Differential Pulse Voltammetry

The differential pulse voltammetry (DPV) curves in Figure 20 illustrate how the electrochemical response of the system depends on pulse amplitude (*E*_pulse_). Measurements span a potential window from −4 to +4 V. The curves are color-coded from purple (0.1 V) to yellow (1.0 V), revealing a narrow but highly active electrochemical region between −0.5 and 0 V. In this region, peak currents range from approximately 0.4 to 0.55 mA. This behavior likely corresponds to redox processes in the proteinoid matrix, such as amino acid residue oxidation/reduction and metal-centered redox transitions. Lower pulse amplitudes produce sharper peak shapes, which enhance resolution for reversible or quasi-reversible processes. In contrast, higher amplitudes result in broader peak profiles and increased peak currents, which improve signal-to-noise ratios but may reduce redox specificity. The absence of significant current activity beyond ±1 V indicates high electrochemical stability of the system, with minimal risk of electrolyte decomposition or electrode degradation. The summary data in Table S4 complements these observations by quantifying electrochemical parameters across the tested range of *E*_pulse_. Peak currents exhibit a nonlinear dependence on pulse amplitude: they begin at 0.429 mA for 0.1 V, reach a maximum of 0.555 mA at 1.0 V, but dip to 0.404 mA at 0.8 V. This intermediate drop may suggest kinetic limitations or surface saturation effects at midrange voltages. Peak potentials shift from −0.282 to −0.632 V, reflecting changes in electron transfer kinetics, possibly due to double-layer capacitance modulation or adsorption dynamics. The area under the curve (AUC) increases steadily from 0.052 to 0.629 mA·V, indicating enhanced total charge transfer at higher *E*_pulse_ values. Altogether, these results confirm the sensitivity of the proteinoid-based electrochemical system to pulse amplitude, providing important guidance for optimizing charge accumulation and detection efficiency in bioinspired applications.

**Figure 20:**
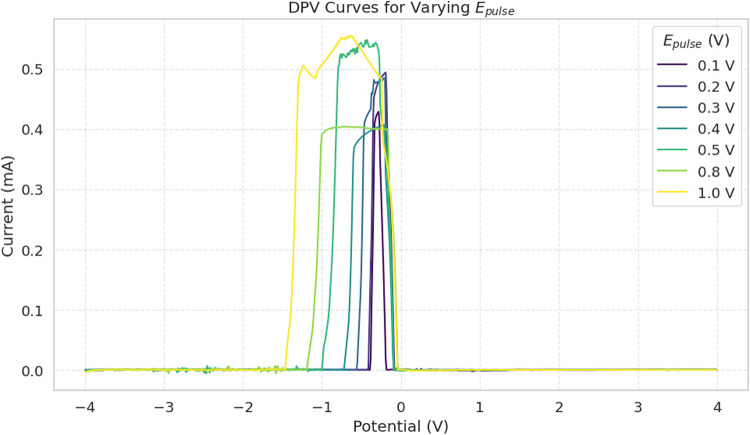
Differential pulse voltammetry (DPV) curves showing the dependence of the electrochemical response of proteinoid-based electrodes on pulse amplitude. Measurements span a potential range from −4 to +4 V, with pulse amplitudes increasing from 0.1 V (purple) to 1.0 V (yellow). All DPV curves exhibit a well-defined electrochemically active window between −0.5 and 0 V, where peak currents increase from ∼0.4 mA at low pulse amplitudes to ∼0.55 mA at the highest amplitude. This region likely corresponds to intrinsic redox processes within the proteinoid matrix, with formal potentials centered near −0.3 V. Lower pulse amplit yield sharper, better-resolved peaks, while higher amplitudes produce broader responses with increased signal intensity. Minimal current outside the ±1 V range indicates electrochemical stability and the absence of parasitic reactions. Overall, the amplitude-dependent DPV behavior highlights the tunability and robustness of proteinoid-based electrodes for bioinspired sensing and electrochemical device applications.

Figure 21 provides a detailed analysis of the area under the curve (AUC) as a function of pulse amplitude (*E*_pulse_), using a quadratic regression model fitted to the experimental data. The fitted trend line is described by eq (eq 35):

35 $$\mathrm{AUC}=0.0725E_{\mathrm{pulse}}^{2}+0.5165E_{\mathrm{pulse}}+0.0070$$

**Figure 21:**
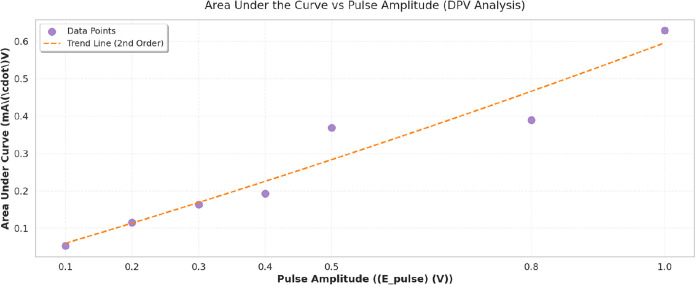
This figure illustrates the relationship between pulse amplitude (*E*_pulse_) and the area under the curve (AUC) in differential pulse voltammetry (DPV) for the olivine-glu_phe_asp proteinoid system. The *E*_pulse_ values range from 0.1 to 1.0 V. The purple scatter markers represent the observed AUC values, while the orange dashed line shows a second-order polynomial fit. The fitted model has the form: AUC = 0.0725 *E*_pulse_^2^ + 0.5165 *E*_pulse_ + 0.0070 with polynomial coefficients [0.0725, 0.5165, 0.0070]. Fit statistics indicate a Residual Sum of Squares (RSS) of 0.0154, a Total Sum of Squares (TSS) of 0.2417, and a coefficient of determination of *R* ^2^ = 0.9363, indicating that the model explains approximately 93.63% of the variance in AUC as a function of *E*_pulse_. The AUC increases nonlinearly, from 0.052 mA·V at 0.1 V to 0.629 mA·V at 1.0 V. This trend suggests enhanced charge transfer or capacitive behavior at higher pulse amplitudes, likely due to improved electrolyte penetration or the activation of additional redox-active sites. The curvature of the quadratic trend indicates the potential onset of a plateau or saturation effect at elevated voltages. This nonlinear behavior is important for optimizing DPV sensitivity in bioinspired sensing and energy storage applications. Careful control of *E*_pulse_ can significantly influence charge transfer efficiency and overall electrochemical performance.

with a coefficient of determination *R* ^2^ = 0.9363, indicating that the model explains approximately 93.63% of the variance in the observed AUC values. The goodness-of-fit metrics, including a residual sum of squares (RSS) of 0.0154 and a total sum of squares (TSS) of 0.2417, further confirm the accuracy of the model in describing the nonlinear behavior of AUC. The increase in AUC becomes more pronounced at higher *E*_pulse_ values, likely due to enhanced capacitive contributions or improved electrolyte penetration into the proteinoid matrix. The quadratic trend also suggests diminishing returns at higher amplitudes, where incremental increases in *E*_pulse_ result in smaller gains in AUC. This may be attributed to the saturation of accessible redox-active sites or kinetic limitations in charge transfer. Such findings highlight the importance of pulse amplitude optimization when designing proteinoid-based electrochemical systems for applications in sensing or energy storage.

The proteinoid system’s response to varying pulse amplitudes (*E*_pulse_) reflects a trade-off between electrochemical sensitivity and signal resolution, as evidenced by the DPV curves and corresponding data in Table S4. Higher *E*_pulse_ values enhance both peak current and the area under the curve (AUC), but also cause peak broadening and shifts in peak potential. This broadening can negatively affect resolution in applications requiring multianalyte detection.

The quadratic AUC fit shown in Figure 21 captures this balance and suggests that an optimal *E*_pulse_ lies in the range of 0.5–0.8 V, where charge transfer is maximized without excessive peak broadening. Theoretically, the peak current in DPV can be approximated by eq (eq 36):

36 $$I_{p}=\mathrm{nFAC}\sqrt{\frac{D}{\pi t_{p}}}\cdot \frac{\Delta E}{2}$$

where *n* is the number of electrons transferred, *F* is the Faraday constant, *A* is the electrode area, *C* is the analyte concentration, *D* is the diffusion coefficient, *t*_p_ is the pulse duration, and Δ*E* corresponds to the pulse amplitude. This relationship supports the observed increase in peak current with *E*_pulse_, reinforcing the empirical trends. However, the fit does not align well with the experimental data (Figure 22): the coefficient of determination is *R* ^2^ < 0.5, and the residual sum of squares is high compared to the total sum of squares. The data points do not follow a linear trend but rather exhibit nonmonotonic behavior, suggesting that other factors may be influencing the response. This deviation reveals the limitations of the simplified DPV model for this system. Possible reasons include nonideal redox kinetics, surface adsorption effects, or capacitive contributions within the proteinoid matrix, especially at higher values of Δ*E*. The electrode diameter is 0.01 mm, corresponding to an area of approximately 7.85 × 10^–11^ m^2^. This gives an estimated value of *nCD* ≈ 3.19 × 10^–8^ mol/m^2^·s, which may not fully account for diffusion or concentration effects in this context.

**Figure 22:**
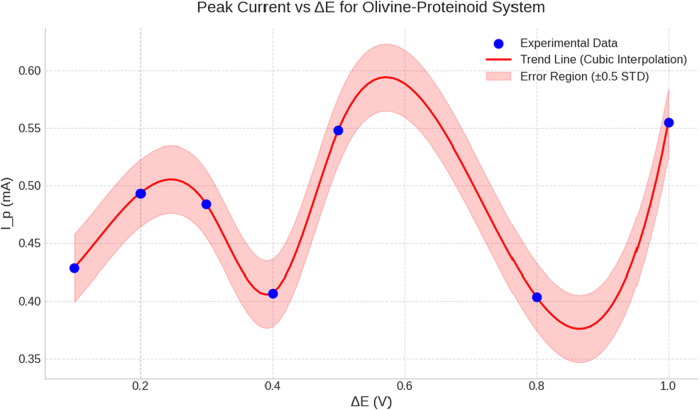
This figure shows how the differential pulse amplitude (Δ*E*) relates to the peak current (*I*_*p*_) in differential pulse voltammetry (DPV) for the olivine_glu_phe_asp proteinoid system. The Δ*E* values range from 0.1 to 1.0 V. The blue scatter markers display the experimental data. Peak currents begin at approximately 0.429 mA at 0.1 V and reach a maximum of 0.555 mA at 1.0 V. There are dips at intermediate voltages, such as 0.407 mA at 0.4 V and 0.404 mA at 0.8 V.

In conclusion, the plot suggests that more advanced models incorporating nonlinear terms are necessary to better describe the electrochemical dynamics of bioinspired materials such as proteinoids. Additionally, optimizing the pulse amplitude Δ*E* could improve sensitivity for future sensing applications. Overall, these findings highlight the suitability of proteinoid-based electrodes for bioinspired electrochemical applications. The system’s concentrated redox activity within a narrow potential window and its tunable response to *E*_pulse_ make it promising for use in sensing, energy storage, or neuromorphic platforms. Future work could explore variations in pulse duration or scan rate to further optimize performance, building on the nonlinear trends identified here to improve both efficiency and selectivity.

To explain the basics of entropy metrics used in this analysis, we begin with Shannon entropy, which quantifies the uncertainty or information content in a discrete random variable *X*, defined by its probability mass function *p*(*x*). Shannon entropy is expressed as eq 37:

37 $$H\left(X\right)=-\sum_{x\in X}p\left(x\right)\log_{2}p\left(x\right)$$

where the summation is taken over all possible states x∈X , and the base-2 logarithm yields entropy in units of bits. This foundational measure is central to defining the entropy rate, which represents the limiting average of the conditional entropy given increasing history. It reflects the average uncertainty per cycle, based on past observations in the discretized peak current series. Similarly, transfer entropy from process *Y* (e.g., peak current) to process *X* (e.g., AUC) is a measure of directed information flow, defined as the conditional mutual information (eq 38):

38 $$TE_{Y\to X}=H\left(X_{t}|X_{t-1},...,X_{t-k}\right)-H\left(X_{t}|X_{t-1},...,X_{t-k},Y_{t-1},...,Y_{t-l}\right)$$

where *k* and *l* are the embedding dimensions (history lengths) of *X* and *Y*, respectively. This formulation captures the reduction in uncertainty about the future state of *X* due to the past of *Y*, beyond what is already explained by the past of *X* itself. In our custom setup, these metrics were applied to discretized (binned) data derived from the time series of peak current and AUC. The results showed that the system exhibits low randomness (low entropy rate) and no detectable cross-parameter causality (zero transfer entropy), highlighting independent evolution of parameters and strong temporal coherence in the system’s response. The cyclic voltammetry data from the olivine–proteinoid sample show a clear adaptive electrochemical response over 100 cycles. There is an exponential decay in peak absolute current, stabilizing around 58.43 μA. This can be modeled as an exponential decay function (eq 39):

39 $$\left|I_{\mathrm{peak}}\right|=1531.22\times e^{-0.1889\times \mathrm{cycle}}+58.43$$

This pattern suggests system memory and conditioning, resembling the complex transfer functions observed in proteinoid microspheres, which emerge due to structural variability. The high lag-1 autocorrelation of 0.9545 and low entropy rate of 0.0766 bits indicate strong temporal predictability and efficient information transfer from prior states. These features support the conceptualization of proteinoids as protoneural networks capable of retaining and distributing electrochemical “memory” through spiking activity.

The transfer entropy from |*I*_peak_| to the area under the curve (AUC) is zero (0.0000 bits), indicating no directional causal influence. This implies that peak current dynamics evolve independently of total charge transfer.

In studying the electrochemical behavior of the olivine–proteinoid system, we assess information transfer using entropy-based metrics derived from information theory. These metrics quantify the predictability and directional flow of data across successive voltage cycles.

The entropy rate of the peak current time series is calculated as 0.0766 bits. This value reflects the average amount of new information generated per cycle. A low entropy rate implies that future states are highly predictable from past values, indicating strong memory retention and reduced uncertainty within the system.

To assess directional causality, we compute transfer entropy from the peak current to the area under the curve (AUC) (Figure 23). The resulting value is 0.0000 bits, indicating that past values of peak current offer no predictive power for future AUC values. In information-theoretic terms, a transfer entropy of zero signifies no directional influence, implying that the parameters evolve independently.

**Figure 23:**
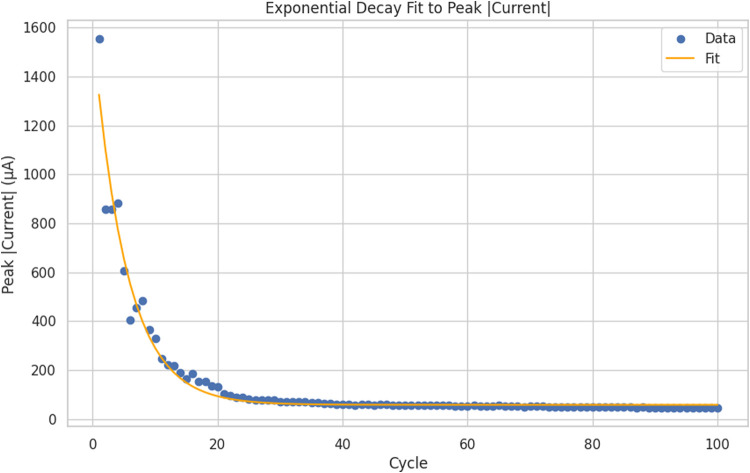
Exponential decay was fitted to the peak absolute current (|*I*_peak_| in μA). This analysis was conducted across 100 voltage cycles in the cyclic voltammetry of an olivine–proteinoid sample. The blue dots represent experimental data, which exhibit a rapid initial decline followed by stabilization. This behavior is well captured by the fitted curve (orange line), described by the model: |*I*_peak_| = 1531.22 × *e* ^–0.1889 × cycle^ + 58.43 The correlation between |*I*_peak_| and the area under the curve (AUC) is moderately positive, with Pearson’s *r* = 0.5730 and a significance of *p* < 0.0001. Lag-1 autocorrelation of |*I*_peak_| is high at 0.9545, indicating strong temporal predictability across cycles. The entropy rate of the time series is low, calculated at 0.0766 bits, suggesting that the system retains significant memory from previous states and exhibits high information transfer over time. Transfer entropy from |*I*_peak_| to AUC is zero (0.0000 bits), indicating no measurable directional information flow between these two parameters. These metrics collectively imply that the system exhibits adaptive behavior, likely due to electrode conditioning or internal material stabilization. While the evolution of current response is highly predictable, the underlying charge dynamics (as reflected in the AUC) appear to evolve independently.

These interpretations align with Shannon’s framework, in which information reduces uncertainty. In the context of this system, the metrics reveal that the proteinoid sample exhibits adaptive behavior and memory-like dynamics, independently of charge accumulation metrics such as AUC.

In a prebiotic context, such behavior could mimic primitive proto-metabolic systems occurring on mineral surfaces such as olivine. Electrochemical adaptation in this setting may have enabled rudimentary information processing without requiring tightly coupled charge integration. These findings suggest that proteinoid microspheres, especially in the presence of substrates like olivine, may serve as viable models for information transfer mechanisms potentially relevant to the origin of life under hydrothermal-like conditions.

The quadratic scaling of the integrated signal with pulse amplitude primarily reflects enhanced charge accumulation and field-driven ionic redistribution within the proteinoid–mineral interface. Such behavior is consistent with capacitive polarization of heterogeneous organic–mineral systems and does not by itself imply computational functionality. Instead, these nonlinear electrochemical responses provide the physical basis for threshold-dependent behavior that can later be interpreted using binary representations. The decrease observed at 0.8 V likely reflects kinetic limitations, such as ion depletion near the interface, diffusion constraints within the proteinoid matrix, or partial surface passivation, which limit further charge accumulation at higher applied potentials.

### Electrochemical Impedance Spectroscopy Analysis of the Olivine-Proteinoid System

The olivine–proteinoid system serves as a compelling model for exploring prebiotic electrochemistry. It builds on the proteinoid theory of the origin of life proposed by Sidney Fox, in which proteinoids form through the thermal polymerization of amino acids. These polymers can self-assemble into microspheres that exhibit membrane-like properties and electrical excitability, potentially resembling early protocells. Olivine, a magnesium–iron silicate mineral common in meteorites and Earth’s mantle, acts in this context as a substrate or catalytic surface. It may mimic conditions found in hydrothermal environments, which are hypothesized to have supported organic synthesis via serpentinization reactions. In our experiment, a galvanostatic time scan was performed at a fixed frequency of 1000 Hz, with no applied DC current (*I*_DC_ = 0 μA). The electrochemical impedance spectroscopy (EIS) response was recorded over approximately 17,012 s (about 4.7 h). This configuration allows the observation of charge transfer processes, interfacial capacitance, and resistance dynamics at the olivine–proteinoid interface. The impedance data, sampled every second, provide a high-resolution timeline of electrochemical parameters. The results indicate a generally stable system with minor variations that may reflect ion transport, adsorption phenomena, or structural changes in the proteinoid–olivine matrix. These findings contribute to our understanding of early bioenergetic models and may also inform the design of bioinspired electrochemical sensors.

Figure 24, subplot (a), shows the impedance magnitude (|*Z*|) over time, measured in ohms. The raw data points, plotted in blue, exhibit noisy fluctuations around a mean of approximately 18.879 Ω. A dark blue rolling mean trend line, computed over a 100-point window, smooths these variations and reveals a gradual decline from about 19.5 to 17.5 Ω. This downward trend suggests evolving interfacial resistance, likely due to processes such as hydration, ion diffusion, or swelling of proteinoid structures on the olivine surface. The light blue error bands represent ±1 standard deviation (with σ ≈ 0.390 Ω), and they narrow over time, indicating increased measurement stability or the system approaching equilibrium. This overall trend supports the conclusion that the olivine–proteinoid interface is mechanically and electrochemically robust. The remaining fluctuations are likely attributed to thermal noise or transient electrochemical events, such as brief pore formation in proteinoid microspheres. Compared to pure proteinoid systems described in the literature—where |*Z*| often exhibits large, spiking variations—this system shows reduced variability. This stabilizing effect may arise from interactions between the mineral substrate and the organic matrix, potentially enhancing conductivity through improved ionic or electronic pathways.

**Figure 24:**
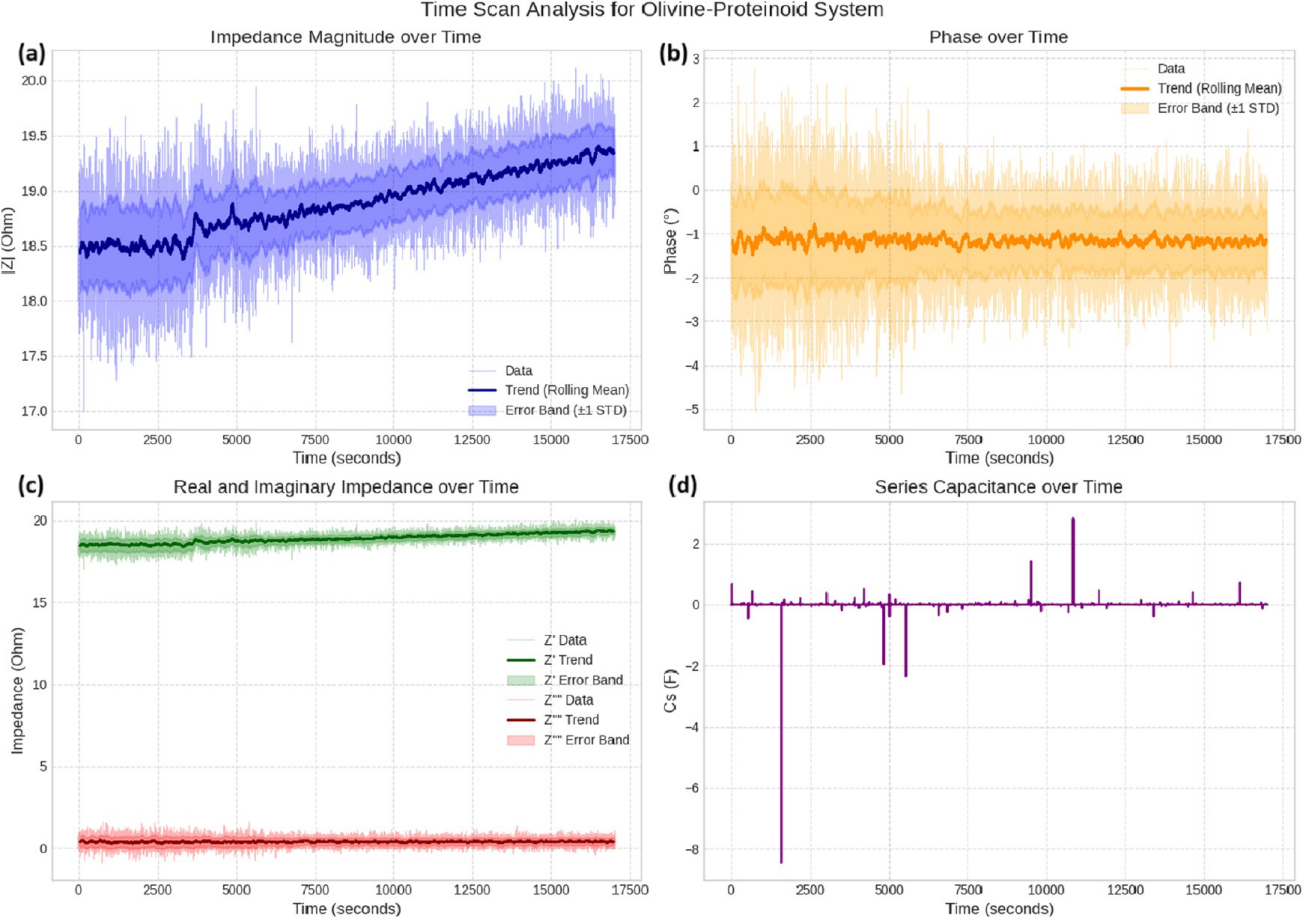
Time Scan Analysis for Olivine–Proteinoid System. The figure consists of four subplots: (a) Impedance magnitude (|*Z*|) over time, showing raw data in blue, a rolling mean trend in dark blue, and error bands (±1σ) in light blue. This highlights the stability and small fluctuations in impedance. (b) Phase over time, displaying raw data in orange, the rolling mean in dark orange, and error bands (±1σ) in light orange, indicating phase variations. (c) Real (*Z*′) and imaginary (*Z*″) components of impedance over time. Raw data are shown in green and red, rolling means in dark green and dark red, and error bands (±1σ) in light green and light red. This reveals the separate contributions to total impedance. (d) Series capacitance (*C*_s_) over time, plotted in purple, showing significant negative spikes likely due to transient system responses. This analysis provides insight into the stability and dynamic behavior of the olivine–proteinoid interface throughout the duration of the experiment.

Figure 24b shows the phase angle over time, measured in degrees. The raw data, plotted in orange, fluctuates around a mean value of −1.166°, indicating predominantly resistive behavior with a slight capacitive contribution (as implied by the negative phase). The dark orange rolling mean reveals a gradual shift from approximately 0 to −2° over the duration of the experiment. The light orange error bands represent ±1 standard deviation (with σ ≈ 0.839°) and capture the temporal variability. Notably, the noise is highest at the beginning and diminishes over time. This early phase variability may stem from initial instabilities such as electrode settling or the hydration of the proteinoid matrix. The overall phase shift suggests a slow buildup of capacitive effects at the olivine–proteinoid interface, possibly due to electrical double-layer formation or charge accumulation on the olivine surface. While proteinoids alone are known to generate action potential-like spiking behavior, the relatively stable phase observed here implies that the presence of olivine modulates the electrical response. Specifically, it appears to suppress inductive elements and favor resistive pathways. This stabilization could support models of steady-state proton or electron transfer in prebiotic systems, where consistent, nonspiking electrical behavior may have been advantageous for early energy transduction mechanisms.

Subplot (c) presents the real (*Z*′) and imaginary (*Z*″) components of the impedance over time. The real part, *Z*′, shown in green, has a mean value of approximately 18.873 Ω, closely matching the overall impedance magnitude |*Z*|, which indicates that the system is primarily resistive. The imaginary component, *Z*″, shown in red, averages around 0.384 Ω, further supporting this resistive character.

Rolling mean trends, shown in dark green and dark red for *Z*′ and *Z*″ respectively, reveal a slight decline in *Z*′ over time, while *Z*″ remains low but consistently positive. This is in agreement with the convention for capacitive systems, where impedance is defined as *Z* = *Z*′ – *jZ*″. The associated error bands represent ±1 standard deviation: approximately 0.391 Ω for *Z*′ and 0.275 Ω for *Z*″. The tighter confidence interval for *Z*′ implies more stable resistive behavior, whereas the broader variation in *Z*″ suggests greater sensitivity to transient phenomena, such as gas bubble formation, ion binding, or microscale interfacial shifts.

This distinction supports the interpretation that the real component arises largely from bulk conductivity through the proteinoid–olivine matrix, while the imaginary component reflects dynamic interfacial capacitance. These patterns may be indicative of charge separation processes relevant to early metabolic functions in prebiotic environments.

Subplot (d) displays the series capacitance (*C*_s_) over time, plotted in purple. Most values cluster around a median of 3.66 × 10^–4^ F, but several pronounced negative spikes reach as low as −8.447 F. These sharp dips are likely artifacts, potentially resulting from measurement noise, abrupt phase changes, or sudden shifts in conductivity during the experiment. Positive capacitance values are consistent with energy storage at the interface, likely due to interactions between polar groups in the proteinoid matrix and the silicate lattice of the olivine surface. Notably, *C*_s_ exhibits significantly more volatility than other measured parameters, with a high standard deviation of 7.45 × 10^–2^ F. This dynamic behavior may be linked to reversible processes such as swelling of proteinoid microspheres or desorption events at the mineral–organic interface. Unlike the stability observed in purely inorganic electrodes, the hybrid behavior observed here underscores the complex, bioinspired nature of the olivine–proteinoid system. These features may prove useful in the development of next-generation capacitive sensors or energy storage devices that mimic primitive bioelectrochemical processes.

The Table 4 supports the figure by providing a statistical summary of the data, comprising all 17,012 measurements collected during the experiment. These values confirm that the system was sampled continuously at a fixed frequency of 1000 Hz. Key metrics—such as the mean impedance magnitude |*Z*| of 18.879 Ω and a standard deviation of 0.390 Ω—quantify the trends observed in subplot (a). The interquartile range (25–75%) spans from 18.634 to 19.152 Ω, reflecting the system’s reliability and low variability. Phase statistics, corresponding to subplot (b), show a mean of −1.166°, with values ranging from a minimum of −5.070° to a maximum of 2.782°. This distribution supports the observed slight negative phase shift, indicating predominantly resistive behavior with minor capacitive contributions. The extremes in series capacitance (*C*_s_), shown in subplot (d), range from −8.447 to 2.833 F. These explain the large spikes seen in the plot. The mean capacitance is reduced to 1.49 × 10^–4^ F, heavily influenced by these outliers. These statistical summaries also facilitate comparisons with previous electrochemical impedance spectroscopy (EIS) studies on proteinoids. In particular, the integration of olivine appears to stabilize the real impedance component *Z*′, which averages 18.873 Ω, and to reduce the variability in the imaginary component *Z*″, which has a standard deviation of 0.275 Ω. This behavior suggests improved interfacial kinetics, potentially enhancing applications in astrobiology, prebiotic chemistry, or organic–inorganic nanomaterial development. While experiments were performed under fixed excitation conditions, future studies will explore the response to varying external inputs (pulse amplitude, frequency, waveforms) to evaluate whether the system can support stimulus-dependent computational behavior.

**Table 4:** Summary Statistics of the Impedance Measurements for the Olivine-Proteinoid System during a Galvanostatic Time Scan at 1000 Hz^a^

	freq (Hz)	neg. phase (deg)	Idc (μA)	|*Z*| (Ohm)	*Z*′ (Ohm)	*Z*″ (Ohm)	*C*_s_ (F)	phase (deg)	mod |*Z*| (Ohm)
count	17,012	17,012	17,012	17,012	17,012	17,012	17,012	17,012	17,012
mean	1000.00	1.166	0.000	18.879	18.873	0.384	1.49 × 10^–4^	–1.166	18.879
std	0.000	0.839	0.000	0.390	0.391	0.275	7.45 × 10^–2^	0.839	0.390
min	1000.00	–2.782	0.000	16.990	16.990	–0.884	–8.447	–5.070	16.990
25%	1000.00	0.624	0.000	18.634	18.629	0.206	2.50 × 10^–4^	–1.716	18.634
50%	1000.00	1.165	0.000	18.904	18.899	0.384	3.66 × 10^–4^	–1.165	18.904
75%	1000.00	1.716	0.000	19.152	19.146	0.566	5.95 × 10^–4^	–0.624	19.152
max	1000.00	5.070	0.000	20.114	20.110	1.630	2.833	2.782	20.114

a The table presents key statistical metrics (count, mean, standard deviation, minimum, 25th percentile, median, 75th percentile, and maximum) for variables including frequency (fixed at 1000 Hz), negative phase, DC current (fixed at 0 μA), impedance magnitude |*Z*|, real part *Z*′, imaginary part *Z*″, series capacitance *C*_s_, phase, and modulus of *Z*. Units are incorporated in the column headers for clarity.

To understand the roles of organic and mineral parts, we must look at how proteinoid assemblies and the olivine substrate affect impedance dynamics. Proteinoid microspheres generate the main electroactive network. They support charge redistribution and dynamic electrical responses. This happens because of their mixed polypeptide structure and ionic functional groups. The olivine substrate offers a mineral interface that affects ion availability. This includes the release of Mg^2+^ and Fe^2+^. It also influences surface adsorption processes and local electrochemical conditions. The proteinoid–olivine system shows how minerals and organic materials work together. Here, the mineral helps stabilize and adjust the electrical behavior of the proteinoid network. It is not just the source of the impedance changes we see. Future work will include systematic control experiments involving proteinoid-only suspensions, mineral-only systems, alternative substrates, and pH-controlled electrolytes to further isolate the contribution of each component.

## Conclusion

This study demonstrates that proteinoid–olivine systems exhibit self-organization, primitive cellular behaviors, and computational capabilities that bridge prebiotic chemistry and bioinspired computing. Olivine templating guided proteinoid assembly into diverse architectures—from spherical microspheres to dendritic networks resembling neural structures. Budding reproduction and hierarchical organization suggest that early cellular division and multicellular coordination may emerge spontaneously from amino acid chemistry in appropriate mineral environments. The appearance of neuron-like branching patterns implies that neural network formation might arise naturally from fundamental physical principles rather than requiring complex biological machinery. Electrochemical characterization revealed stable impedance profiles supporting Boolean logic operations (AND, OR, XOR, NOT) through threshold-based switching. Galvanostatic measurements revealed spontaneous oscillations. These showed burst dynamics and non-Poissonian statistics. These features are signs of complex feedback mechanisms, which are ideal for reservoir computing. The olivine substrate provided both structural templating and electrochemical stabilization, creating a dynamic mineral–organic interface that may reflect conditions in early Earth hydrothermal systems where life potentially originated. These findings illuminate pathways toward sustainable bioinspired technologies. The demonstrated logic operations, memory-like behavior, and network formation suggest applications in neuromorphic computing, adaptive sensing, and soft robotics. Self-assembly and self-repair capabilities could enable robust systems combining biological adaptability with electronic functionality. Future investigations should explore scalability, learning capacity, and complex task processing in these systems. The mineral–organic interface appears central to understanding both the origins of biological computation and the development of advanced biohybrid technologies. Can the principles governing proteinoid–olivine systems reveal universal mechanisms underlying the transition from geochemistry to biochemistry—and ultimately, to cognition?

## Supplementary Material



## Data Availability

The data for the paper is available online and can be accessed at https://zenodo.org/records/16423245.

## Notes

Complete cyclic voltammetry analysis for all 100 consecutive voltage cycles including minimum and maximum potential values, current ranges, peak current magnitudes with corresponding potentials, and area under curve calculations for olivine-proteinoid electrochemical characterization (Table S1); summary statistics of impedance magnitude, phase angle, and real and imaginary components obtained from galvanostatic impedance spectroscopy over 30 cycles, illustrating the temporal evolution and stabilization of the electrical properties of olivine–proteinoid samples (Table S2); results of Boolean logic gate operations applied to binary-thresholded impedance data from galvanostatic impedance spectroscopy, demonstrating how temporal impedance variations in olivine–proteinoid samples can be mapped to logical states and transitions relevant to bioinspired computing (Table S2); proteinoid microspheres in olivine acid solution undergo a time-dependent morphological transition from smooth, uniform spheres to complex neuron-like structures (Figure S1); and summary of the differential pulse voltammetry results for the olivine–glu_phe_asp proteinoid system over pulse amplitudes from 0.1 to 1.0 V (Table S4) (PDF)
