# Supplementary material for: Proteinoid Computing on Olivine Substrates
Source: Langmuir. 2026 Mar 27;42(13):9595–625. doi: 10.1021/acs.langmuir.6c00952 (PMC13068363; doi:10.1021/acs.langmuir.6c00952)
Supplement: Supplementary file 1 [file la6c00952_si_001.pdf]

Supporting Information

Proteinoid Computing on Olivine Substrates

Panagiotis Mougkogiannis[1,*] and Andrew Adamatzky[1]

[1]Unconventional Computing Laboratory, University of the West of England, Bristol, UK, BS16 1QY

Email: Panagiotis.Mougkogiannis@uwe.ac.uk

The electrochemical characterization of the proteinoid–olivine system reveals complex computational functionality across multiple measurement modalities, as summarized in Table S1, Table S2, Table S3, and Table S4, and supported by the morphologies shown in Figure S1. Cyclic voltammetry over 100 cycles (Table S1) demonstrates strong electrochemical conditioning and stability. Peak currents decrease from 1553.37 μA to a stable range of 45–60 μA, while peak potentials shift from -0.5 V to -0.25 V, indicating surface reorganization and electrical double-layer equilibration. The area under the curve rapidly decays from 178.85 μA·V to stable values of 5–9 μA·V, confirming steady-state behavior suitable for repeatable computational operations. Galvanostatic impedance spectroscopy at 1000 Hz over 30 cycles (Table S2) reveals a three-phase temporal evolution. The mean impedance magnitude increases from 0.072 kΩ to a maximum of 0.162 kΩ at t = 6291 s, before stabilizing near 0.140 kΩ by t = 20,267 s. The mean phase angle rises from 11.4° to 17.0° during interfacial buildup and relaxes to 14.2° at steady state, reflecting adaptive capacitive behavior. These dynamics enable Boolean logic operations (Table S3) via threshold binarization at 0.14 kΩ, with sustained AND activation during the impedance plateau and XOR responses marking state transitions at t = 4196 s and t = 20,267 s. Differential pulse voltammetry (Table S4) shows strong sensitivity to pulse amplitude E_{pulse}. Peak currents increase from 0.429 mA at 0.1 V to 0.555 mA at 1.0 V, with a non-monotonic decrease at 0.8 V indicative of kinetic saturation. Peak potentials shift cathodically from -0.282 V to -0.632 V, while the area under the curve increases quadratically from 0.052 to 0.629 mA·V (R^2 = 0.936), consistent with enhanced charge accumulation at higher excitation. These electrochemical behaviors are directly associated with the neuron-like architectures shown in Figure S1. Structural motifs including soma-like compartments, axon-like conduits, dendritic elaborations, and multi-node connectivity provide the physical substrate for signal propagation and computation. The network exhibits long-range conduits (10–25 μm), highly connected nodes (degree 4–5), and fractal organization (D_f = 2.03), supporting distributed information processing. The progressive dendritic development visible in Figure S1 correlates with the improved electrochemical stability observed in Table S1, demonstrating the tight coupling between structural maturation and computational function in proteinoid–olivine systems.

Table S1: cyclic voltammetry analysis of electrochemical behavior across 100 consecutive voltage cycles.

Index	Cycle	Min Potential (V)	Max Potential (V)	Min Current (μA)	Max Current (μA)	Peak —Current— (μA)	At Potential (V)	AUC (μA·V)
0	1	-0.5	0.4995	-1553.3743	811.8896	1553.3743	-0.5	178.8464
1	2	-0.5	0.4995	-857.142	297.2733	857.142	-0.43	21.0013
2	3	-0.5	0.4995	-859.0493	230.7543	859.0493	-0.42	-16.9992
3	4	-0.5	0.4995	-881.3176	290.631	881.3176	-0.42	-20.525
4	5	-0.5	0.4995	-606.6584	375.1125	606.6584	-0.4	3.1714
5	6	-0.5	0.4995	-404.6718	327.1187	404.6718	-0.37	9.4691
6	7	-0.5	0.4995	-454.0771	339.0826	454.0771	-0.37	18.9155
7	8	-0.5	0.4995	-483.498	346.0349	483.498	-0.38	5.267
8	9	-0.5	0.4995	-363.9688	304.4641	363.9688	-0.36	4.4139
9	10	-0.5	0.4995	-329.1882	267.5139	329.1882	-0.35	3.1587
10	11	-0.5	0.4995	-246.5615	224.2502	246.5615	-0.3401	5.2515
11	12	-0.5	0.4995	-220.0922	220.0255	220.0922	-0.3301	9.1432
12	13	-0.5	0.4995	-216.9259	201.5717	216.9259	-0.3301	6.0906
13	14	-0.5	0.4995	-174.3395	188.1774	188.1774	-0.2101	7.8446
14	15	-0.5	0.4995	-148.8572	163.3436	163.3436	-0.2201	7.6183
15	16	-0.5	0.4995	-184.3483	183.9811	184.3483	-0.3201	9.0803
16	17	-0.5	0.4995	-154.3599	152.4859	154.3599	-0.3201	8.4463
17	18	-0.5	0.4995	-134.4805	153.8402	153.8402	-0.2201	9.0374
18	19	-0.5	0.4995	-129.5929	137.0745	137.0745	-0.2201	7.9128
19	20	-0.5	0.4995	-104.1535	131.8818	131.8818	-0.2301	11.1384
20	21	-0.5	0.4995	-86.6202	103.6099	103.6099	-0.2401	10.7603
21	22	-0.5	0.4995	-78.5997	94.5596	94.5596	-0.2401	10.2318
22	23	-0.5	0.4995	-74.394	89.9819	89.9819	-0.2401	9.7559
23	24	-0.5	0.4995	-71.0896	90.2585	90.2585	-0.2401	9.5839
24	25	-0.5	0.4995	-66.6311	82.6863	82.6863	-0.2401	9.2863
25	26	-0.5	0.4995	-65.129	79.6297	79.6297	-0.2401	8.9612
26	27	-0.5	0.4995	-67.2414	79.6631	79.6631	-0.2401	8.1093
27	28	-0.5	0.4995	-64.6808	78.7094	78.7094	-0.2401	8.7039
28	29	-0.5	0.4995	-66.1304	79.0432	79.0432	-0.2401	8.8514
29	30	-0.5	0.4995	-60.766	72.6345	72.6345	-0.2401	8.4963
30	31	-0.5	0.4995	-59.1924	71.4472	71.4472	-0.2401	8.2943
31	32	-0.5	0.4995	-57.9717	70.3934	70.3934	-0.2401	8.51
32	33	-0.5	0.4995	-56.9274	70.8464	70.8464	-0.2401	8.3778
33	34	-0.5	0.4995	-56.0786	69.2585	69.2585	-0.2401	8.0262
34	35	-0.5	0.4995	-53.1556	66.0827	66.0827	-0.2401	8.0901
35	36	-0.5	0.4995	-51.3961	65.7775	65.7775	-0.2401	7.6108
36	37	-0.5	0.4995	-49.7355	62.253	62.253	-0.2401	7.4414
37	38	-0.5	0.4995	-49.8518	62.3097	62.3097	-0.2401	7.2323
38	39	-0.5	0.4995	-48.2368	61.0127	61.0127	-0.2401	7.1983
39	40	-0.5	0.4995	-47.5678	60.0889	60.0889	-0.2401	7.135
40	41	-0.5	0.4995	-46.6899	59.0541	59.0541	-0.2401	7.178
41	42	-0.5	0.4995	-46.1568	58.296	58.296	-0.2401	7.0239
42	43	-0.5	0.4995	-45.4544	60.1149	60.1149	-0.2501	7.0344
43	44	-0.5	0.4995	-46.4849	59.0367	59.0367	-0.2401	6.9168
44	45	-0.5	0.4995	-46.8888	57.5998	57.5998	-0.2401	6.7436
45	46	-0.5	0.4995	-44.8398	58.9709	58.9709	-0.2501	6.5948
46	47	-0.5	0.4995	-44.6858	58.9814	58.9814	-0.2501	6.5233
47	48	-0.5	0.4995	-44.1927	57.7431	57.7431	-0.2501	6.5492
48	49	-0.5	0.4995	-43.1527	57.2185	57.2185	-0.2501	6.4508
49	50	-0.5	0.4995	-43.3397	57.0612	57.0612	-0.2501	6.4836
50	51	-0.5	0.4995	-43.5013	56.6301	56.6301	-0.2501	6.4351
51	52	-0.5	0.4995	-42.9205	56.0703	56.0703	-0.2501	6.4047
52	53	-0.5	0.4995	-43.8627	56.6516	56.6516	-0.2501	6.2833
53	54	-0.5	0.4995	-42.262	55.5916	55.5916	-0.2501	6.3135
54	55	-0.5	0.4995	-42.9	56.2534	56.2534	-0.2501	6.3232
55	56	-0.5	0.4995	-43.0597	55.7217	55.7217	-0.2501	6.2252
56	57	-0.5	0.4995	-41.8848	54.9507	54.9507	-0.2501	6.1867
57	58	-0.5	0.4995	-41.2316	53.9355	53.9355	-0.2501	6.0709
58	59	-0.5	0.4995	-40.8587	53.3857	53.3857	-0.2501	6.1187
59	60	-0.5	0.4995	-41.7299	54.5764	54.5764	-0.2501	6.2
60	61	-0.5	0.4995	-42.9229	55.1476	55.1476	-0.2501	6.2638
61	62	-0.5	0.4995	-42.098	54.4419	54.4419	-0.2501	6.0388
62	63	-0.5	0.4995	-40.1959	52.4597	52.4597	-0.2501	6.0201
63	64	-0.5	0.4995	-40.7304	53.7486	53.7486	-0.2501	5.962
64	65	-0.5	0.4995	-43.4646	55.0399	55.0399	-0.2501	5.9447
65	66	-0.5	0.4995	-40.5969	52.236	52.236	-0.2501	6.0699
66	67	-0.5	0.4995	-39.6918	52.0763	52.0763	-0.2501	6.0617
67	68	-0.5	0.4995	-39.7529	52.1974	52.1974	-0.2501	5.9552
68	69	-0.5	0.4995	-39.173	51.157	51.157	-0.2501	6.1465
69	70	-0.5	0.4995	-39.3795	51.8732	51.8732	-0.2501	5.9636
70	71	-0.5	0.4995	-40.1324	52.2751	52.2751	-0.2501	6.0303
71	72	-0.5	0.4995	-39.9388	51.8069	51.8069	-0.2501	6.0018
72	73	-0.5	0.4995	-39.1268	51.167	51.167	-0.2501	6.0282
73	74	-0.5	0.4995	-39.0777	51.2132	51.2132	-0.2501	6.0125
74	75	-0.5	0.4995	-39.0429	50.6224	50.6224	-0.2501	5.9773
75	76	-0.5	0.4995	-38.7701	50.3988	50.3988	-0.2501	5.8271
76	77	-0.5	0.4995	-38.4373	49.52	49.52	-0.2501	5.7692
77	78	-0.5	0.4995	-37.9972	49.3836	49.3836	-0.2501	5.7486
78	79	-0.5	0.4995	-37.9595	49.3335	49.3335	-0.2501	5.6294
79	80	-0.5	0.4995	-37.5165	48.13	48.13	-0.2501	5.6262
80	81	-0.5	0.4995	-37.5179	48.6969	48.6969	-0.2501	5.6965
81	82	-0.5	0.4995	-37.6181	48.8743	48.8743	-0.2501	5.6449
82	83	-0.5	0.4995	-37.4731	48.1958	48.1958	-0.2501	5.6148
83	84	-0.5	0.4995	-37.4111	48.1247	48.1247	-0.2501	5.5723
84	85	-0.5	0.4995	-37.3181	47.7361	47.7361	-0.2501	5.5507
85	86	-0.5	0.4995	-37.2838	47.8386	47.8386	-0.2501	5.5838
86	87	-0.5	0.4995	-37.279	47.604	47.604	-0.2501	5.5929
87	88	-0.5	0.4995	-37.157	47.9917	47.9917	-0.2501	5.5635
88	89	-0.5	0.4995	-37.176	47.573	47.573	-0.2501	5.3918
89	90	-0.5	0.4995	-36.7311	46.2713	46.2713	-0.2501	5.3525
90	91	-0.5	0.4995	-36.8375	46.235	46.235	-0.2501	5.3844
91	92	-0.5	0.4995	-36.7784	46.1454	46.1454	-0.2501	5.3786
92	93	-0.5	0.4995	-36.692	46.2279	46.2279	-0.2501	5.3116
93	94	-0.5	0.4995	-36.5457	45.9861	45.9861	-0.2501	5.3181
94	95	-0.5	0.4995	-36.5843	45.7367	45.7367	-0.2501	5.3467
95	96	-0.5	0.4995	-36.5728	45.3481	45.3481	-0.2501	5.4096
96	97	-0.5	0.4995	-36.4322	44.9709	44.9709	-0.2501	5.4198
97	98	-0.5	0.4995	-36.3277	45.6476	45.6476	-0.2501	5.33
98	99	-0.5	0.4995	-36.4436	45.9894	45.9894	-0.2501	5.3749
99	100	-0.5	0.4995	-36.2715	45.8383	45.8383	-0.2501	[illegible]

Table S2: Descriptive statistics for impedance measurements over 30 cycles in the galvanostatic impedance spectroscopy test on olivine–proteinoid samples are summarized as follows. The mean impedance magnitude ($|Z|$) starts at $0.072\,\mathrm{k\Omega}$ at $t = 0\,\mathrm{s}$, peaks at approximately $0.162\,\mathrm{k\Omega}$ around $t = 6291\,\mathrm{s}$, and then gradually decreases to $0.140\,\mathrm{k\Omega}$ by $t = 20267\,\mathrm{s}$. This behavior may reflect initial charging or interfacial reactions, followed by stabilization. The standard deviations of $|Z|$ follow a similar trend, indicating increased variability during the peak phase. Mean phase angles rise from 11.35° to approximately 17°, before stabilizing near 15°, suggesting a shift in the capacitive characteristics of the system. The maximum real and negative imaginary impedance components (Z' and $-Z''$) reach peak values of $0.877\,\mathrm{k\Omega}$ and $0.394\,\mathrm{k\Omega}$, respectively. Overall, these metrics highlight the temporal evolution of the system's electrical properties during the experiment.

**Cycle**	**Time (s)**	**Mean $|Z|$ (kΩ)**	**Std $|Z|$ (kΩ)**	**Mean Phase (°)**	**Std Phase (°)**	**Max Z' (kΩ)**	**Max $-Z''$ (kΩ)**
0	0.000000	0.072182	0.078957	11.354273	17.970817	0.313465	0.139475
1	702.900000	0.074581	0.085296	12.015469	18.207636	0.331601	0.140379
2	1403.000000	0.077047	0.089937	12.347906	18.666843	0.338084	0.160904
3	2101.500000	0.082277	0.101223	13.133771	19.682732	0.368767	0.193334
4	2799.600000	0.090316	0.118163	14.040544	20.340094	0.454396	0.253191
5	3497.400000	0.123264	0.179399	16.027860	23.178782	0.661395	0.321791
6	4195.700000	0.146934	0.222259	16.911283	23.873520	0.772578	0.393853
7	4894.300000	0.155246	0.236778	16.985905	23.760571	0.843810	0.375319
8	5593.000000	0.160342	0.245141	16.714895	23.979111	0.859965	0.363121
9	6291.500000	0.161903	0.245243	16.605736	23.642398	0.877228	0.345564
10	6990.200000	0.160580	0.240175	16.400490	23.394006	0.854161	0.333843
11	7689.000000	0.159483	0.235781	15.838693	23.470047	0.853600	0.323352
12	8387.700000	0.159021	0.232833	15.795206	23.252847	0.804524	0.318872
13	9086.500000	0.158676	0.230503	15.692921	23.075070	0.821467	0.311770
14	9785.300000	0.157388	0.227205	15.579055	22.919561	0.772824	0.297669
15	10484.100000	0.156975	0.225940	15.291300	22.892264	0.794876	0.299564
16	11182.800000	0.153520	0.217942	15.438046	22.539967	0.756075	0.284158
17	11881.600000	0.151619	0.214385	15.087886	22.585576	0.752851	0.273693
18	12580.400000	0.150391	0.211673	15.428113	22.119878	0.755245	0.265135
19	13279.200000	0.148096	0.206607	15.074163	22.228839	0.707819	0.261282
20	13978.000000	0.147658	0.205529	14.890200	22.227359	0.695174	0.253514
21	14677.000000	0.145828	0.201561	14.967812	21.994282	0.695128	0.250051
22	15375.500000	0.144226	0.198710	14.830214	21.988858	0.695357	0.244447
23	16074.400000	0.143220	0.196173	14.916404	21.704324	0.687655	0.241228
24	16773.200000	0.142328	0.194046	14.732658	21.628834	0.675996	0.234757
25	17472.000000	0.141062	0.190908	14.762466	21.598121	0.649946	0.234858
26	18170.600000	0.141609	0.192219	14.595007	21.710188	0.660323	0.233844
27	18869.600000	0.141034	0.191914	14.646033	21.678251	0.668136	0.230945
28	19568.100000	0.140611	0.190676	14.146166	21.845763	0.659036	0.229342
29	20267.000000	0.139957	0.189548	14.176171	21.800927	0.645909	0.228231

Table S3: This table shows the results from using Boolean logic gates on binary-thresholded impedance data. The data originates from galvanostatic impedance spectroscopy on olivine–proteinoid samples. The mean impedance magnitude $|Z|$ is set at a threshold of 0.14 kΩ. If the value is 0.14 kΩ or higher, it is assigned a binary 1 (logic high), indicating higher resistance, likely due to interfacial buildup. If it is below 0.14 kΩ, it receives a 0 (logic low), corresponding to lower resistance. Inputs A and B represent consecutive binary values—A being the current value and B the previous one (via shift)—thus simulating temporal logic operations on the system's electrical evolution. The outputs for the AND, OR, XOR, NAND, NOR, and NOT gates show distinct patterns. For instance, the AND gate remains high (1) from $t = 4894.3$ s to $t = 19568.1$ s, matching the impedance plateau. The XOR gate detects transitions at $t = 4195.7$ s and $t = 20267.0$ s. The NOT, NAND, and NOR gates reveal logical inversions, marking the deactivation phases. This digital mapping demonstrates the promise of proteinoid materials in bio-inspired computing. Here, impedance changes function analogously to logic switching events over time.

**Time (s)**	**Mean $\|Z\|$ (kΩ)**	**Binary Input**	**Input A**	**Input B**	**AND Output**	**OR Output**	**XOR Output**	**NAND Output**	**NOR Output**	**NOT Output (on A)**
0.0	0.072182	0	0	0	0	0	0	1	1	1
702.9	0.074581	0	0	0	0	0	0	1	1	1
1403.0	0.077047	0	0	0	0	0	0	1	1	1
2101.5	0.082277	0	0	0	0	0	0	1	1	1
2799.6	0.090316	0	0	0	0	0	0	1	1	1
3497.4	0.123264	0	0	0	0	0	0	1	1	1
4195.7	0.146934	1	1	0	0	1	1	1	0	0
4894.3	0.155246	1	1	1	1	1	0	0	0	0
5593.0	0.160342	1	1	1	1	1	0	0	0	0
6291.5	0.161903	1	1	1	1	1	0	0	0	0
6990.2	0.160580	1	1	1	1	1	0	0	0	0
7689.0	0.159483	1	1	1	1	1	0	0	0	0
8387.7	0.159021	1	1	1	1	1	0	0	0	0
9086.5	0.158676	1	1	1	1	1	0	0	0	0
9785.3	0.157388	1	1	1	1	1	0	0	0	0
10484.1	0.156975	1	1	1	1	1	0	0	0	0
11182.8	0.153520	1	1	1	1	1	0	0	0	0
11881.6	0.151619	1	1	1	1	1	0	0	0	0
12580.4	0.150391	1	1	1	1	1	0	0	0	0
13279.2	0.148096	1	1	1	1	1	0	0	0	0
13978.0	0.147658	1	1	1	1	1	0	0	0	0
14677.0	0.145828	1	1	1	1	1	0	0	0	0
15375.5	0.144226	1	1	1	1	1	0	0	0	0
16074.4	0.143220	1	1	1	1	1	0	0	0	0
16773.2	0.142328	1	1	1	1	1	0	0	0	0
17472.0	0.141062	1	1	1	1	1	0	0	0	0
18170.6	0.141609	1	1	1	1	1	0	0	0	0
18869.6	0.141034	1	1	1	1	1	0	0	0	0
19568.1	0.140611	1	1	1	1	1	0	0	0	0
20267.0	0.139957	0	0	1	0	1	1	1	0	1

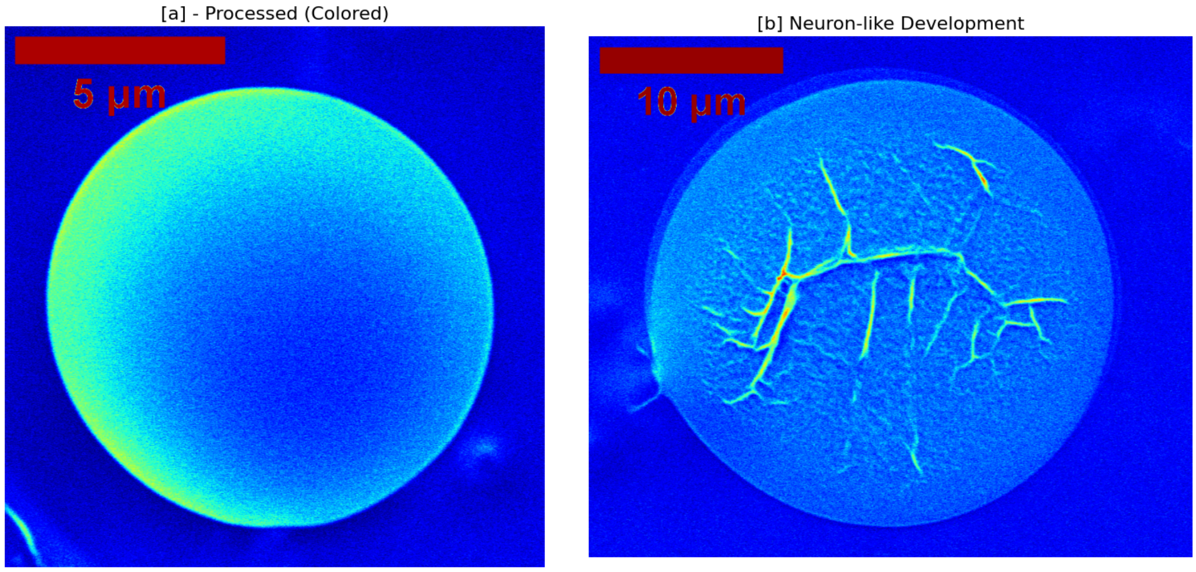

Figure S1: Proteinoid microspheres change over time, showing neuron-like shapes and forming dendritic networks in olivine acid solution. This suggests a different way to develop cellular complexity, not just through simple budding. **Panel [a] - Processed (Colored):** A mature microsphere (6 μm diameter) shows a uniform spherical shape. It has a smooth surface and a consistent internal structure. The green-to-blue color gradient from core to edge highlights this. This lack of surface bumps or internal structure shows that it is in a pre-differentiation state. Here, the proteinoid matrix is balanced with low surface energy. The color shifts from yellow-green in the center to blue at the edges show a density change. This change matches the formation of a hydrophobic core and a hydrophilic surface. It follows the classic proteinoid self-assembly model. Here, the total free energy, ΔG_{total}, is the sum of hydrophobic, electrostatic, and surface energies. This total reaches its lowest point. **Panel [b] - Neuron-like Development:** This advanced stage shows a dramatic change. It has a complex branched shape, about 15 μm in total span. There are also many dendritic-like projections coming from the central soma. The yellow-green lines show high-density proteinoid channels. They create a network with branches that are 2–8 μm long and about 0.5–1 μm wide. This branching pattern looks like neuronal dendrites. It has a clear hierarchy with primary, secondary, and tertiary branches. Bifurcation points indicate growth that is guided by chemical gradients or mechanical stress. The network topology shows fractal-like features. The branching density follows the rule $N_{\text{branches}} \propto L^{D_f}$. Here, D_f is the fractal dimension, and L is the characteristic length scale. The blue background matrix keeps its structure. The yellow network shows areas with more proteinoids. This might mean there are phase separations or sol-gel changes inside the microsphere. This neuron-like morphogenesis shows a new way to reproduce. Unlike budding, which creates separate offspring, the parent structure changes inside. It develops specialized functional areas. The dendritic structure hints at early signal transmission and network connections. Each branch may act as a pathway for information in a basic neural network. This change in shape can happen due to local pH, ionic strength, or mechanical stress. These factors can change the uniform spherical shape. They also promote growth in certain directions due to crystallography or chemical gradients. Simple proteinoid chemistry can create complex structures. This suggests that neural-like architectures might have developed on their own in prebiotic systems. These structures could be an early step in forming biological neural networks. **Scale bars:** 5 μm (panel a), 10 μm (panel b).

Table S4: Differential pulse voltammetry (DPV) results for the olivine–glu_phe_asp proteinoid system across pulse amplitudes (E_{pulse}) from 0.1 to 1.0 V. Reported parameters include peak current (mA), peak potential (V), and area under the curve (AUC, $\text{mA} \cdot \text{V}$). Peak current shows a non-linear dependence on E_{pulse}, increasing from 0.4286 mA at 0.1 V to 0.5547 mA at 1.0 V, with a notable decrease at 0.8 V, suggesting possible kinetic or surface effects. The peak potential shifts progressively from -0.282 V to -0.632 V, indicating changes in redox dynamics at higher excitation. AUC increases steadily from 0.0525 to 0.6294 mA·V, reflecting enhanced total charge transfer or capacitive contributions. Overall, the system exhibits strong sensitivity to pulse amplitude, highlighting its tunability for bio-inspired sensing and energy storage applications.

**index**	$E_{\textbf{pulse}}(V)$	**Peak Current (mA)**	**Peak Potential (V)**	**Area Under Curve (mA·V)**
0	0.1	0.429	-0.282	0.052
1	0.2	0.494	-0.192	0.115
2	0.3	0.484	-0.212	0.164
3	0.4	0.407	-0.212	0.193
4	0.5	0.548	-0.432	0.369
5	0.8	0.404	-0.682	0.390
6	1.0	0.555	-0.632	0.629
